# Structural parameters of palindromic repeats determine the specificity of nuclease attack of secondary structures

**DOI:** 10.1093/nar/gkab168

**Published:** 2021-03-27

**Authors:** Anissia Ait Saada, Alex B Costa, Ziwei Sheng, Wenying Guo, James E Haber, Kirill S Lobachev

**Affiliations:** School of Biological Sciences and Institute for Bioengineering and Bioscience, Georgia Institute of Technology, Atlanta, GE 30332, USA; School of Biological Sciences and Institute for Bioengineering and Bioscience, Georgia Institute of Technology, Atlanta, GE 30332, USA; School of Biological Sciences and Institute for Bioengineering and Bioscience, Georgia Institute of Technology, Atlanta, GE 30332, USA; School of Biological Sciences and Institute for Bioengineering and Bioscience, Georgia Institute of Technology, Atlanta, GE 30332, USA; Department of Biology and Rosenstiel Basic Medical Sciences Research Center, Waltham, MA 02454-9110, USA; School of Biological Sciences and Institute for Bioengineering and Bioscience, Georgia Institute of Technology, Atlanta, GE 30332, USA

## Abstract

Palindromic sequences are a potent source of chromosomal instability in many organisms and are implicated in the pathogenesis of human diseases. In this study, we investigate which nucleases are responsible for cleavage of the hairpin and cruciform structures and generation of double-strand breaks at inverted repeats in *Saccharomyces cerevisiae*. We demonstrate that the involvement of structure-specific nucleases in palindrome fragility depends on the distance between inverted repeats and their transcriptional status. The attack by the Mre11 complex is constrained to hairpins with loops <9 nucleotides. This restriction is alleviated upon RPA depletion, indicating that RPA controls the stability and/or formation of secondary structures otherwise responsible for replication fork stalling and DSB formation. Mus81-Mms4 cleavage of cruciforms occurs at divergently but not convergently transcribed or nontranscribed repeats. Our study also reveals the third pathway for fragility at perfect and quasi-palindromes, which involves cruciform resolution during the G2 phase of the cell cycle.

## INTRODUCTION

Palindromic sequences or inverted repeats (IRs) are a potent source of chromosomal breakage and rearrangements in bacteria and many eukaryotic organisms because they can adopt hairpin and cruciform structures. In bacteria, perfect and quasi-palindromes (IRs separated by nonrepeated spacer) in plasmid or phage DNA are frequently deleted with the length of the spacer being the major factor for their instability (reviewed in (1)). When present on the *Escherichia coli* chromosome, IRs cause DSB formation and large inverted duplications (2). In budding and fission yeast and in mice, IRs have been shown to strongly induce ectopic and allelic recombination, crossing-over, and a variety of gross chromosomal rearrangements (GCRs) including deletions, translocations and gene amplification (e.g. (3–10)). In humans, palindromic sequences have been found at breakpoints of chromosomal translocations and are implicated in the pathogenesis of diseases. For example, palindromic AT-rich repeats (PATRRs) are responsible for the most frequent, recurrent non-Robertsonian translocation t(11;22)(q23;q11), which causes Emanuel syndrome, as well as nonrecurrent translocations (11). Palindrome-mediated large deletions, interchromosomal insertions and translocations also cause several types of ϵγΔβ thalassemia (12), X-linked congenital hypertrichosis syndrome (13) and hereditary renal cell carcinoma (14). Finally, palindromes promote oncogene amplification (15–22).

Several nucleases have been implicated in the attack of hairpin or cruciform structures leading to the genetic instability of IRs. One major player in the metabolism of both palindromes and quasi-palindromes is the prokaryotic SbcCD nuclease and its eukaryotic homolog Mre11-Rad50, part of the Mre11 complex (Mre11-Rad50-Xrs2/Nbs1) (23). In *E. coli*, SbcCD induces breakage at IRs in a replication-dependent manner. SbcCD targets hairpins formed by quasi-palindromes on the lagging strand template leading to a two-ended DSB that can be repaired by recombination with the sister chromatid (24,25). In contrast, perfect palindrome cleavage by SbcCD leads to cell death presumably because of hairpin formation on both the leading and lagging strands, thus eliminating the templates amenable to repair (26). In fission yeast, the recombinogenic effect of a 160 bp palindrome is dependent on the Mre11 complex. It has been postulated that the recombinogenic DSB is generated by the nuclease activity of Mre11 (3,4). However, in *Saccharomyces cerevisiae*, the Mre11 complex is not involved in breakage at a large IR consisting of two Ty1 elements with a ∼280 bp spacer in strains where DNA polymerase δ was down-regulated (27). Previously, we demonstrated that in *S. cerevisiae* the Mre11 complex does not generate DSBs at a closely spaced *Alu* quasi-palindrome (*Alu*-QP), but it is required, along with Sae2, for opening and resection of breaks that have hairpin-capped ends (6,28). This disparity on the effect of the Mre11 complex on DSB generation at IRs might be attributed to the differences in the formation or stability of hairpins with different loop sizes during replication and their availability for nuclease attack. This surmise is experimentally addressed in this study.

Several structure-specific endonucleases (SSEs) were described as a responsible for generating DSBs at IRs in eukaryotes. A genetic-based assay in budding yeast identified Rad1/Rad10 as a contributor to chromosomal fragility at a short IR (29). Mus81/Mms4 acting together with Rad1/Rad10 and Slx1/Slx4 was found to be involved in causing fragility at the AT-rich Flex1 sequence motif located on a yeast artificial chromosome (30). However, Nag *et al.* showed that Rad1/Rad10 is neither required for generating nor for processing DSBs at a 140 bp IR in budding yeast (31). Mus81/Mms4 was also shown to cleave plasmid-born cruciform structures formed by PATTRs in budding yeast (32). In cancer cell lines, MUS81/EME1 attacks secondary structures formed by AT-rich microsatellites upon depletion of the WRN helicase (33). Inagaki *et al.* found that GEN1 (homologue of yeast Yen1) can cleave cruciforms formed by PATTRs in a plasmid transfection assay in human cells (34). However, Mus81/Mms4 is not involved in recombination induced by either quasi-palindromes (28) or palindromes (35). Yen1 is not required for fragility at Flex1 and is not responsible for palindrome-induced intra-chromosomal recombination (30,35). This rather diverse spectrum of effects could be explained by the fact that (i) repeats used in these studies had different structural characteristics; (ii) IRs were located on plasmids versus chromosomes; (iii) whether the conclusions about the effect of the SSEs were based only on biological assays or were accompanied by direct detection of DSBs.

In this study, we examined the effect of SSEs on IR-mediated chromosomal DSB formation in budding yeast using a set of structurally different palindromic sequences. The strength of our study is the use of a combination of experimental approaches that include: (i) a sensitive assay for GCR induction and (ii) direct physical detection of chromosomal breaks and replication intermediates. We found that perfect and quasi-palindromes with spacers <9 bp can efficiently block DNA replication and are targets for MRX/Sae2 attack, a mechanism reminiscent of SbcCD attack on hairpins in bacteria. The specificity of MRX/Sae2 activity depends on the formation or stability of hairpins since RPA depletion leads to replication arrest and Sae2-dependent breaks in strains containing *Alu*-QP with spacers >8 bp. Besides the spacer length, transcription is another important factor affecting palindrome fragility and specificity of the involved nucleases. We observed that Mus81/Mms4 contributes to DSB formation at divergently transcribed *URA3* or *HIS4* IRs. However, Mus81/Mms4 and other known SSEs (Yen1, Slx1/Slx4, Rad1/Rad10 and Mlh1/Mlh3) do not generate breaks at the nontranscribed *Alu* and *IS50* palindromes. Interestingly, we found that MRX/Sae2- and Mus81/Mms4-independent breakage at palindromes is not replication-dependent which points towards cruciform formation and resolution by an unknown nuclease during the G2 phase. These results suggest that chromosomal fragility at palindromic sequences is caused by the interplay of multiple nucleases at different stages of the cell cycle and is dependent on the type of secondary structure formed. Our data define parameters of palindrome attack by nucleases and extend our understanding of modalities of DSBs formation at IRs to elucidate the poorly defined mechanisms underlying palindrome-associated chromosomal aberrations and human diseases.

## MATERIALS AND METHODS

### Strains, plasmids and oligonucleotides

Yeast strains and oligonucleotides used in this study are listed in Supplementary Tables S3 and S4, respectively. The strains used in this study are isogenic and based on *MATα bar1*Δ *his7–2 trp1*Δ *ura3*Δ *leu2–3, 112 ade2*Δ *lys2*Δ *cup1*Δ *yhr054c*Δ *cup2*Δ *V34205::ADE2lys2::IR V29616::CUP1*. The GCR cassette composed of the counter selectable markers *CUP1*, *CAN1* and *ADE2* (in this order from telomere to centromere) is located on the left arm of chromosome V. IRs are located within *lys2* that is telomere-distal to the GCR cassette. For all experiments, freshly thawed yeast strains were used. All strains were grown at 30°C and manipulated using standard yeast genetics methods. The media used in this study are YPD, YPR (1% yeast extract, 2% peptone and 2% dextrose of raffinose), uracil-drop out synthetic medium and histidine-drop out synthetic medium supplemented with 2 mM 3-AT. Target nonessential genes were disrupted using one of the following drug-resistance or prototrophic markers: *kanMX* (kanamycin), *hphMX* (hygromycin), *natMX* (nourseothricin) or *TRP1* (tryptophan). The essential gene *RFA2* was placed under control of a *tetO7* repressible promoter to create the strain TET-*RFA2* and expression was downregulated by adding doxycycline to the media (6). All IRs were inserted into *LYS2* which had been placed telomere-distal to the GCR cassette (*V34205)*. Strains containing *Alu* perfect palindrome (*Alu*-PAL) and *Alu* palindromes interrupted by spacers (*Alu*-QPs) were generated by the *delitto perfetto* approach (36). A CORE cassette containing the markers *kanMX* and *URA3* was inserted at the center of symmetry of the *Alu* inverted repeat. The CORE cassette was then replaced by transformation with an oligomer (Supplementary Table S4) containing homology to the flanking *Alu* repeats separated by the *Swa*I palindromic restriction site to create *Alu*-PAL. Similarly, *Alu*-QPs were generated by transformation with oligomers (Supplementary Table S4) containing asymmetrical spacers ranging from 5 to 12 bp flanked by homology to the *Alu* repeats. Transformants were selected on synthetic media lacking adenine and containing 1 g/l of 5-fluoroorotic acid (5-FOA). Uracil auxotroph and kanamycin-sensitive clones were selected and tested for the presence of the expected *Alu*-PAL or *Alu*-QP by Southern blot. Genomic DNA was extracted and digested with *Bst*EII to reveal replacement of the CORE cassette and with *Bst*EII and *Sfa*NI (or *Bsm*I, whichever enzyme corresponded to the restriction site in the asymmetrical spacer) to reveal the presence of the spacer. *LYS2-*specific probes located upstream and downstream of the *Alu* repeats were used to reveal the resulting fragments. Strains containing *IS50*-PAL (37) *URA3*- and *HIS4*-PAL (38) were created as previously described. pRSafe_CRE-EBD (gift from Gartenberg’s lab) was integrated into *LEU2* to build conditional quasi-palindrome strains.

### GCR and mutagenesis rate estimation by fluctuation test

Strains were grown on YPD agar media for 3 days at 30°C for most of the experiments. *URA3*-PAL strains were grown on YPD or uracil-drop out medium. *HIS4*-PAL strains were grown on YPD and single colonies were cultured overnight in histidine-drop out medium supplemented with 2 mM of 3-AT. TET-*RFA2* strains were grown on YPD with 62.5 μg/l of doxycycline. For each strain 12–24 independent colonies were selected for a fluctuation test (39). Appropriate dilutions of cells were plated on YPD and canavanine-containing plates (arginine-drop out medium containing a low amount of adenine (4 mg/l) and 60 mg/l of L-canavanine). White and red colonies grown on canavanine-containing media reflect CAN1 mutagenesis and GCR events, respectively. GCR and mutation rates were calculated as previously described (28).

### DSB detection

Cells from overnight cultures were embedded in 0.75% low-melting agarose plugs at a concentration of 25 × 10^8^ cells/ml. Each plug (∼4 × 10^8^ cells/plug) was treated with 0.5 mg of zymolyase and 1 mg of proteinase K. Around one-fourth of a plug (∼40 μl) was used per sample for DSB detection either by separating chromosomes with contour-clamped homogeneous electric field (CHEF) electrophoresis or restriction digestion prior to standard gel electrophoresis. Each plug was equilibrated in the electrophoresis buffer prior to gel casting. The broken left arm of chromosome V (∼43 kb) was separated from intact chromosome V (∼580 kb) by CHEF for 26 h (or 28 h, Figure 4A) in a 1% PFGE certified agarose gel in 0.5× TBE at 14°C and 6 V/cm with an included angle of 120°, an initial switching time 3.14 s and final switching time of 7.68 s (or 12.56 and 17.53 s, respectively, Figure 4A). For restriction digestion of DNA, the plugs were washed twice in 1× TE (10 mM Tris-Cl, pH 8.0, 0.1 mM EDTA), once in 1× TE containing 1 mM of PMSF, once in water and equilibrated in 2× and then 1× restriction buffer. Each plug was digested with 50 units of *Afl*II, *Bgl*II or *Bsr*GI overnight. Digested plugs containing DNA were loaded in 1% (*Afl*II digestion) or 0.8% (*Bgl*II and *Bsr*GI digestions) agarose gels and run in 1× TBE for 18 and 20 h, respectively. The agarose gels containing the separated DNA fragments were treated consecutively with 0.25 N HCl, alkaline buffer (1.5 M NaCl, 0.5 M NaOH) and neutralization buffer (1.5 M NaCl, 1 M Tris, pH7.5). DNA was then transferred to a charged nylon membrane in 10× SSC for 2 h at 70 mmHg via Posiblotter (Stratagene). Southern hybridization was performed using a ^32^P-radiolabeled *HPA3-* or *LYS2*-specific probe in PerfectHyb Plus Hybridization Buffer at 68°C overnight. The membranes were washed twice in a buffer containing 0.1× SSC and 0.1% SDS at 68°C and were exposed to a phosphor storage screen. Densitometry analysis of the broken fragments was performed using ImageJ software (NIH).

### Replication intermediate analysis by 2DGE

Strains were inoculated in 400 ml of YPD and grown overnight. When OD600 reached 0.8, the cells were synchronized in G1 for 3 h with 0.1 μg/ml of alpha factor. For TET-*RFA2* strains, 2 μg/ml of doxycycline was added to the medium to downregulate RPA. After washing the cells twice with water, cells were released into fresh YPD containing 12.5 μg/ml of pronase E. Fifty minutes post release, sodium azide was added to a final concentration of 0.1% and the cells were collected in the presence of 0.2 M frozen EDTA pH8 (20 mM final). Genomic DNA was extracted by the standard cesium chloride method using the protocol ‘Joel Huberman’s DNA isolation procedure with modifications by Bonny Brewer’ (https://fangman-brewer.genetics.washington.edu/DNA_prep.html) and DNA was processed as described in (40). DNA was digested with 50 units of *Afl*II for several hours and precipitated in ethanol. For the first dimension, restriction digestion fragments were separated in a 0.4% agarose gel and run in 1× TBE at 1.7 V/cm for 22 h. For the second dimension, gel slices from the first dimension containing the fragment of interest, were loaded into a 1.2% agarose gel containing 0.3 mg/l of ethidium bromide. Gel electrophoresis was performed in 1X TBE containing 0.3 mg/ml of ethidium bromide at 6 V/cm for 10 h at 4°C. The gels were processed for Southern hybridization as described in the previous section. A *LYS2*-specific probe, corresponding to the ARS-proximal side of the IR, was used to highlight replication intermediates.

### DSB formation across the cell cycle (conditional IR)

Cells were grown overnight in YPR supplemented with 300 mg/l G418. At an OD600 of 0.8, the cultures were either synchronized in G1 with 0.1 μg/ml of alpha factor or in G2/M with 15 μM nocodazole or 500 nM 1-NM-PP-1. For each time point, the volume of culture necessary to make a plug was transferred into a falcon tube containing sodium azide (0.1% final) and frozen 0.2 M EDTA pH8 (20 mM final). The first aliquot was taken after 3 h of synchronization and represents the condition (-) for Cre induction in the figures. Galactose (2% final) and estradiol (4 μM final) were added to the remaining cultures to induce Cre expression and additional alpha-factor was added to the G1 arrested cells. After 3 h of induction, an aliquot of cells was collected for FACS analysis and to make plugs. This time point is represented by the condition (+) for Cre induction for the G2/M arrested cells (with nocodazole or 1-NM-PP-1) and 0 min for the G1 arrested cells. To release G1 arrested cells into S phase, the remainder of the culture was washed twice with water and resuspended in fresh YPD containing 12.5 μg/ml of pronase E. Samples were taken 20, 40, 50, 70 and 90 min after release. Plugs were made and DSBs were revealed as described in the section ‘DSB detection.’ Plugs were digested either with *Afl*II or *Bsr*GI to detect the ARS-proximal or telomere-proximal side of the break, respectively, using the corresponding *LYS2*-specific probe.

### Flow cytometry

For each time point, 1 ml of cells was harvested and fixed in cold 70% ethanol. An aliquot was collected by centrifugation and resuspended in 1 ml of 50 mM Tris, pH 8.0, 15 mM NaCl containing 2 mg/ml of RNase A. After 2 h of RNase treatment at 37°C, 25 μl of 20 mg/ml Proteinase K was added and the samples were incubated at 55°C for an additional hour. DNA was stained with 1× SYTOX green in 50 mM Tris pH 7.5. The samples were sonicated briefly and DNA content was measured using an Accuri C6 (Bio-Rad).

### RT-qPCR

At least three independent colonies from each strain were inoculated in YPD and grown overnight. Cells were washed once with water and were diluted to an OD600 of 0.1 in the appropriate medium. *URA3*-PAL strains were cultured in YPD (+ Uracil condition) and uracil-drop out medium (- Uracil condition) and *HIS4*-PAL strains were cultured in YPD (+ Histidine condition) and histidine-drop out medium supplemented with 2 mM of 3-AT (- Histidine + 3-AT condition). After 4 h of growth, RNA was isolated using an Aurum Total RNA Kit (BIO-RAD) with the DNaseI treatment step lengthened to 45 min at 37°C. The absence of gDNA was confirmed by PCR using the extracted RNA as a template. Reverse transcription and RT-qPCR were performed using a Luna Universal One-Step RT-qPCR Kit (New England BioLabs) on a QuantStudio 6 Flex Real-Time PCR system (Applied Biosystems). Primers used are listed in Supplementary Table S4. mRNA levels were normalized to *ACT1* to determine relative abundance (41). Significance was estimated by two sample *t* tests.

### Quantification and statistical analysis

GCR and mutagenesis rate: All data presented are the median of the rates for at least 12 biological replicates and a 95% confidence interval is indicted (39).

DSB detection: For each graph, the nature of the values and errors bars is mentioned in the figure legends. All data presented are the average of at least three biological replicates. Bands were visualized by phosphorimaging (Typhoon, GE Healthcare) and quantified with ImageJ software. Percent of DSB corresponds to the DSB signal normalized to the corresponding unbroken fragment/chromosome V. Data were analyzed and graphed with Prism8. Statistical analysis was performed using a *t* test. When error bars correspond to the 95% confidence interval, statistics are not indicated.

## RESULTS

### Experimental system to study DSB formation at palindromic repeats

**Figure 1.**
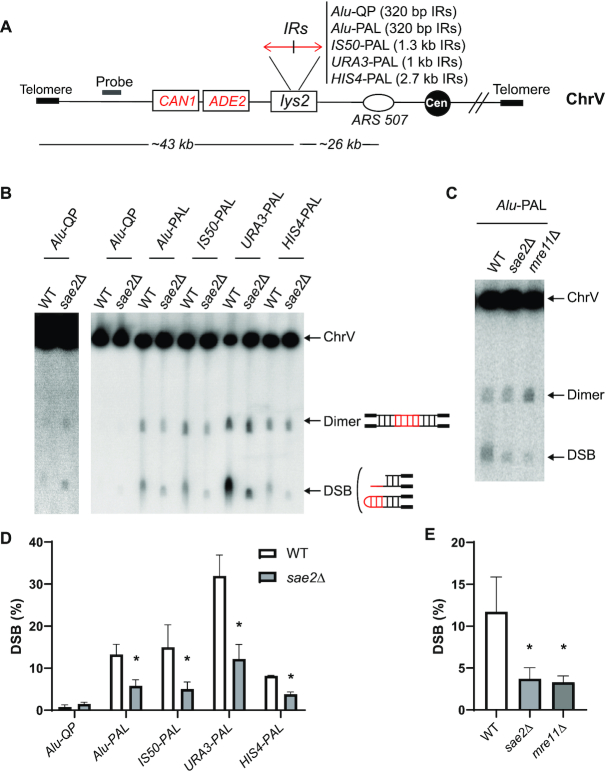
DSBs at perfect but not quasi palindromes are partly induced by Sae2/MRX. (**A**) Scheme of IRs located on chromosome V. Different palindromes cloned into *LYS2* have been inserted on the left arm of chromosome V, ∼26 kb away from *ARS507*. Secondary structures at IRs lead to the formation of hairpin-capped breaks (∼43 kb), duplication of which leads to the formation of an inverted dimer twice the break size (∼86 kb). DSBs can be revealed using Southern blot and hybridization after chromosome separation by CHEF. *HPA3* probe (gray rectangle) is used to highlight the different chromosomal fragments. IRs with the corresponding size of the repeat are indicated. (**B**) Representative image of DSB detection at different IRs in WT and *sae2*Δ. Genomic DNA embedded in agarose plugs was separated by CHEF. Chromosomal fragments (indicated by black arrows) correspond to unbroken chromosome V (585 kb), dimer (86 kb) and break (43 kb). Due to a lower DSB frequency at QP compared to perfect palindromes, a higher exposure of *Alu*-QP is presented on the left. (**C**) Representative image of DSB detection at *Alu*-PAL in WT and in the absence of MRX-Sae2 complex. Detection and analysis were performed as in (B). (**D** and **E**) Quantification of DSBs presented in (B) and (C), respectively. Values are the mean of at least three independent biological replicates ± standard deviation (SD); * *P*< 0.02.

The experimental system to monitor DSB formation at IRs is based on the sensitive GCR assay described in (10) (Figure 1A). Briefly, the 43 kb region between telomere and *CAN1* on chromosome V does not contain any essential genes and can be lost without affecting cell viability. *ADE2* is placed between *CAN1* and the IR to differentiate between DSB-mediated arm loss (leading to GCRs) and *CAN1* mutations. GCR isolates are canavanine-resistant red (Ade^-^) colonies, while mutations in *CAN1* give rise to canavanine-resistant white colonies. The spontaneous rate of arm loss in control strains without repeats, is extremely low: 3 × 10^–9^/division. Four different repeats were inserted in inverted orientation into *LYS2*: 320 bp *Alu*, 1.3 kb *IS50*, and actively transcribed 1.1 kb *URA3* and 2.7 kb *HIS4*. Repeats are either separated by a spacer (abbreviated as -QP) or have no spacer (abbreviated as -PAL). Overall, insertion of interrupted or perfect palindromes next to *CAN1* causes a dramatic (>10 000-fold) increase in the GCR rate (Table 1). The deduced mechanism for IR-induced GCRs is the formation of hairpin-capped breaks, that after DNA synthesis, result in dicentric dimers, followed by breakage in anaphase and repair involving break-induced replication using a non-homologous chromosome as a template (10).

**Table 1.** Effect of palindromic sequences on GCRs in WT and *sae2*Δ

	GCR rate (×10^−7^)	
*lys2-inserted repeat*	WT	*sae2*Δ
**No repeat** ^a,b^	0.03 (0.02–0.04)^c^	0.4 (0.3–0.6)
***Alu*-QP** ^b^	617 (559–691)	1343 (1134–1717)
***Alu*-PAL** ^b^	7000 (5017–9943)	7829 (6560–9459)
***IS50-*PAL**	8600 (4190–8620)	7200 (5840–8060)
***URA3*-PAL** ^b^	850 (680–960)	13460 (11090–15570)
***HIS4*-PAL**	8620 (8200–9710)	9750 (8700–11220)

^a^Rates previously published (Saini et al., 2013).

^b^Cell plating efficiency was assayed in these strains and was ∼ 95–100%.

^c^Numbers in parenthesis correspond to the 95% confidence interval.

### MRX/Sae2 attacks secondary structures at perfect palindromes but not quasi-palindromes separated by a 12 bp spacer

We have previously shown that *Alu*-QP with a 12 bp spacer located on chromosome II induces hairpin-capped DSBs. We have also found that the Mre11 complex does not trigger DSBs at *Alu*-QP but is required along with Sae2 to process hairpin termini (28). This is consitent with Sae2 being a regulator of the Mre11 endonuclease activity (42). A defect in hairpin opening results in replication of broken fragments and formation of inverted dimers, including a dicentric chromosome. Accordingly, *Alu*-QP-induced GCRs are increased in *mre11*Δ, *rad50*Δ (6,10) and *sae2*Δ (Table 1). The frequency of breakage on chromosome V was similar (∼1%) in WT and *sae2*Δ (Figure 1B), consistent with previous studies of *Alu*-QP-induced DSBs on chromosome II (28). IR-induced DSB formation is always accompanied by the appearance of inverted dimers. In WT, resected broken arms run with delay and show a spread signal compared to unresected broken arms in *sae2*Δ strains (43,44). This explains why DSBs may seem more visible in *sae2*Δ albeit DSB quantification is similar to WT. In strains containing *Alu-*PAL, GCR rates and chromosomal breakage were greatly increased, compared to *Alu*-QP (Table 1 and Figure 1B). When *SAE2* or *MRE11* were disrupted, we observed 50–70% reduction in the *Alu*-PAL-mediated breaks (Figure 1B–E). In these mutants, broken molecules migrated as a discrete band and faster than resected breaks in WT, indicating the presence of unprocessed hairpin termini (43,44). Similarly, the breakage level at *IS50*-PAL, *URA3*-PAL and *HIS4*-PAL ranged from 8 to 30%, and *sae2*Δ caused a 2–3-fold reduction in DSB formation (Figure 1B–D). Remarkably, *URA3*-PAL exhibited the strongest level of fragility: approximately one-third of the WT cells carried broken chromosome V. Nevertheless, viability of these strains was not affected and *URA3*-PAL was stably maintained suggesting recurring breakage and restoration of the palindrome (see ‘Discussion’ section). Notably, in *sae2*Δ strains, high levels of *URA3*-PAL-induced breaks correlated with high levels of GCRs, in contrast to WT (Table 1)

From these observations we can draw several conclusions. First, MRX/Sae2 has a dual role in IR-induced DSBs metabolism: it is responsible for ∼30 to 50% of breaks formed at perfect but not at quasi-palindromes and is required for processing hairpin-capped breaks induced by both types of IR. Notably, despite the strong effect on DSB formation, disruption of *SAE2* in strains with perfect palindromes did not lead to a decrease in GCRs (Table 1), indicating that MRX/Sae2-mediated breaks are not channeled into GCRs in contrast to hairpin-capped breaks. Second, there are at least two pathways for DSBs formation at IRs: an MRX/Sae2-dependent pathway operating only at perfect palindromes and an MRX/Sae2-independent pathway that operates at both types of IR, yet has a stronger effect at perfect palindromes.

### Spacer length determines *Alu*-IR DSB potential, susceptibility to the MRX/Sae2 attack and ability to block replication forks

To identify structural parameters governing IR stability and MRX/Sae2 attack, we generated strains with *Alu*-QPs separated by spacers ranging from 5 to 12 bp. Since even in the case of perfect palindromes 4–5 nt remain unpaired in the hairpin loop due to stereochemical constraints (45,46), *Alu*-QP with 1–4 bp spacers were omitted from the analysis. The spacers contained the nonpalindromic *Sfa*NI restriction site and random nucleotides (Supplementary Figure S1A). We refer to each quasi-palindrome as *Alu*-QP, followed by the spacer length. The impact of each spacer on GCRs and DSBs was assessed in WT and *sae2*Δ.

**Figure 2.**
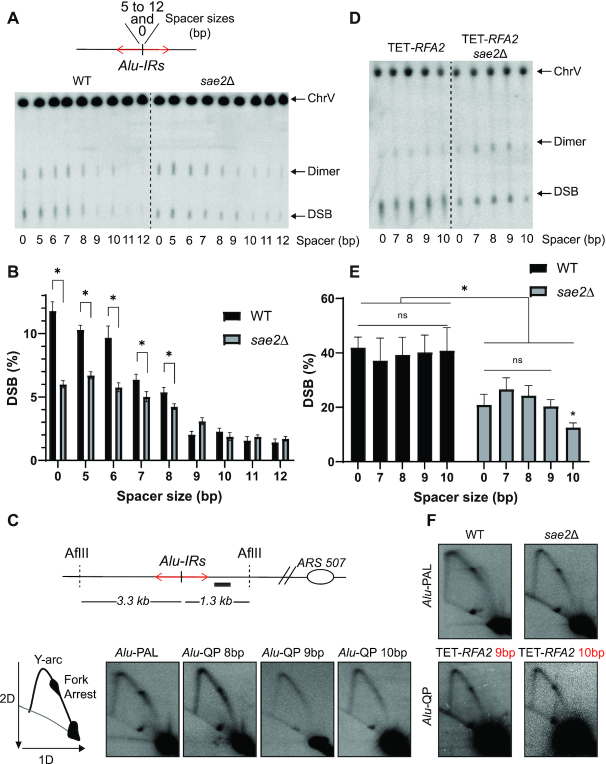
Analysis of the ability of IRs to induce DSBs and replication fork block according to the spacer length. (**A**) Scheme of *Alu*-PAL (0 bp) and *Alu*-IRs separated by 5 to 12 bp asymmetrical spacers (top panel) and representative image of DSB detection at these IRs (bottom panel). Detection was performed as described in Figure 1A and B. (**B**) Quantification of DSBs in (A). Values are the mean of at least six independent biological replicates ± standard deviation (SD); * *P*< 0.02. (**C**) 2D gels analysis of replication intermediates (RIs) in strains containing the indicated *Alu*-IRs. Illustration of restriction digestion with *Afl*II and the position of the probe used to reveal RIs (black rectangle) is depicted on the top. Bottom panel: Representative RIs analysis in the indicated *Alu*-IRs. Scheme of RIs analysis is depicted on the left. (**D**) DSB detection in WT (TET-*RFA2*) and *sae2*Δ (TET-*RFA2 sae2*Δ). Detection was carried out as described in Figure 1A and B. (**E**) Quantification of DSBs in TET-*RFA2* strains (WT and *sae2*Δ); * *P*< 0.04. (**F**) Replication intermediate analysis in WT and *sae2*Δ strains containing *Alu*-PAL and in TET-*RFA2* strains with *Alu*-QP9 and 10.

In WT, DSB levels at *Alu*-QP5 and 6 were only slightly decreased compared to *Alu*-PAL. *Alu*-QP7 and 8 showed a drop of ∼50% in the frequency of breaks (∼5%) whereas *Alu*-QP9 showed a drop of 80% (Figure 2A and B). The length of the spacer had a similar impact in *sae2*Δ. However, the DSB level was reduced in *sae2*Δ, compared to WT, at *Alu*-PAL and *Alu*-QPs5–8 but not at *Alu*-QPs with longer spacers (Figure 2A and B). This indicates that MRX/Sae2 attack occurs at hairpins with loops <9 nt.

GCRs analysis showed the same inverse correlation between the spacer length and fragility, in both WT and *sae2*Δ (Supplementary Table S1). While disruption of *SAE2* in strains with *Alu*-PAL or *Alu*-QPs5–8 did not lead to a significant decrease in GCRs, *Alu*-QPs with spacers ≥9 bp exhibited higher GCR rates in *sae2*Δ compared to WT. We confirmed this observation using *Alu*-QPs with a different intervening sequence containing the nonpalindromic *Bsm*I restriction site (Supplementary Figure S1B), thus excluding an effect due to the sequence composition (Supplementary Table S2). We concluded that MRX/Sae2 plays a role in DSB formation at *Alu*-QP separated by up to 8 bp whereas IRs with longer spacers are insensitive to MRX/Sae2 attack.

Stable secondary structures formed by IRs cause replication fork pausing (6,47). Therefore, we analyzed the impact of spacer length on replication fork progression using 2D gel electrophoresis of replication intermediates. Strains with *Alu*-PAL and *Alu*-QP8 showed a robust fork arrest at the location of the repeats, as evidenced by the accumulation of molecules at one point along the replication arc (Figure 2C; Supplementary Figure S2C and D). Similar to *Alu*-QP12 (6), we did not detect fork pausing in strains harboring *Alu*-QP9 or 10. This shows that MRX/Sae2 attack on secondary structures and their capacity to hinder fork progression are correlated. We propose that stable hairpins on the lagging strand template are formed when IRs are separated by <9 bp. Overall, our data suggest that the length of the spacer is the main structural feature that determines MRX/Sae2 attack and predisposition to strongly block replication, with an 8 bp spacer being the transition point.

### Rfa2 downregulation destabilizes *Alu*-IRs and mitigates spacer effect

Based on the data presented above, we reasoned that *Alu*-PAL and *Alu*-QP may form hairpins in single-stranded DNA (ssDNA) on the lagging strand template with a comparable efficiency, but hairpin loops >8 nt may be targeted by proteins involved in secondary structure dissolution. Replication protein A (RPA) is one of the possible players in this process. RPA is an essential eukaryotic ssDNA-binding protein playing pivotal roles in DNA metabolism. RPA can efficiently bind ssDNA stretches with an 8 nt initial binding site (48,49) and is known to be involved in destabilizing secondary structures such as hairpins (50,51). We previously showed that downregulation of one RPA subunit, Rfa2, increases *Alu*-QP12 fragility (6). The natural *RFA2* promoter was replaced by a *tetO*-repressible promoter to create the strain referred to as TET-*RFA2*. Downregulation was induced by adding doxycycline in the media (6). In TET-*RFA2* strains, that is, upon Rfa2 downregulation, all IRs showed the same high level of DSBs (35–40%) regardless of the spacer length (Figure 2D–E). Surprisingly, DSBs were decreased not only at *Alu*-PAL but also at all *Alu*-QPs in *sae2*Δ TET-*RFA2* (Figure 2D–E). Similar frequencies of breakage were observed in *Alu*-PAL and *Alu*-QP7–9 (18–23%) in *sae2*Δ strains, whereas break level was affected to a greater extent at *Alu*-QP10 (12%).

We noticed that Rfa2 downregulation affected DSB levels in *Alu*-PAL and *Alu*-QP9 and 10 in WT strains to different extents. TET-*RFA2* strains exhibited a 3.6-fold increase in breakage at *Alu*-PAL compared to *RFA2* proficient strains, whereas a ∼20-fold increase was observed at *Alu*-QP9 and 10 (Supplementary Figure S2A). This difference was not as pronounced in *sae2*Δ strains (Supplementary Figure S2B). These data indicate that RPA both stabilizes *Alu*-IRs in general and prevents MRX/Sae2 attack at *Alu*-IRs separated by >8 bp.

We then tested if *Alu*-QP9 and 10 acquire the ability to arrest replication forks when RPA is downregulated. In contrast to WT strains, a strong replication fork arrest was observed at *Alu*-QP9 and 10 in TET-*RFA2* strains (Figure 2F; Supplementary Figure S2C and D).

Altogether, these data suggest that in the absence of RPA, *Alu*-QPs with spacers longer than 8 bp form stable secondary structures that can both arrest replication forks and become targets for MRX/Sae2.

### Breaks occurring at *Alu*-PAL are symmetrical and not affected by known SSEs

**Figure 3.**
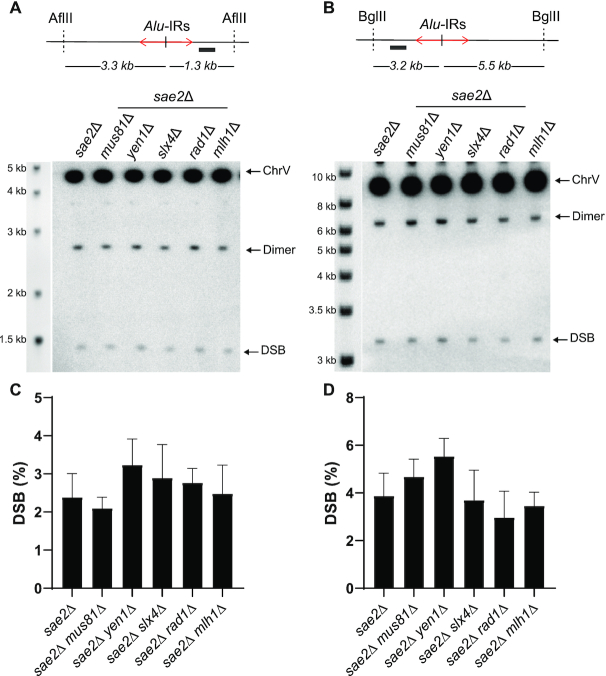
DSBs at *Alu*-PAL are symmetrical and not affected by known SSEs. (**A** and **B**) Detection of the ARS- and telomere proximal DSBs, respectively, formed at *Alu*-PAL in *sae2*Δ strains deleted for the indicated SSE. Genomic DNA embedded in agarose plugs was digested with *Afl*II (A) or with *Bgl*II (B). The relative position of the repeat and the restriction sites are indicated. The solid bar indicates the position of the *LYS2*-specific probes. The bands corresponding to the unbroken fragment, the dimer and the DSB are indicated on the representative images for DSB detection. The DNA ladder, highlighted with ethidium bromide prior Southern blot hybridization, with indicated sizes is shown on the left. (**C** and **D**) Quantification of DSBs presented in (A and B), respectively, relative to unbroken chromosome V. Values are the mean of at least three independent biological replicates ± 95% confidence intervals.

Accumulation of DSBs in *sae2*Δ (Figure 1B and C) demonstrates the existence of another pathway for break formation at perfect palindromes. We analyzed the effect of different SSEs on chromosomal fragility induced by *Alu*-PAL in *sae2*Δ strains. We assayed the involvement of SSEs capable of processing Holliday junctions or other branched DNA structures: Mus81/Mms4, Yen1, Slx4/Slx1, Rad1/Rad10 and Mlh1/Mlh3 (reviewed in (52,53)). GCR analysis revealed that the deletion of *MUS81*, *YEN1*, *SLX4*, *RAD1* or *MLH1* did not significantly affect GCR rates (Supplementary Figure S3A). This indicates that none of these nucleases is solely responsible for MRX/Sae2-independent DSBs induced at *Alu*-PAL. Consistent with the GCR analysis, DSB detection did not reveal involvement of the above SSEs in *Alu*-PAL fragility. DSBs were detected using an ARS- and a telomere-proximal probe upon digestion of agarose embedded chromosomal DNA with *Afl*II and *Bgl*II, respectively (Figure 3A and B, top panels). The size of the fragment corresponding to DSBs occurring at *Alu*-PAL was expected to be 1.3 kb upon *Afl*II digestion and detection using the ARS-proximal probe. A 3.2 kb fragment was expected upon *Bgl*II digestion and detection using the telomere-proximal probe. Replication of hairpin-capped breaks leads to inverted dimers twice the size of the breaks, which are manifested by the appearance of a 2.6 or 6.4 kb fragment upon *Afl*II or *Bgl*II digestion, respectively. The results presented in Figure 3 revealed that DSBs induced at *Alu*-PAL can be detected from both sides of the repeat. Deletion of *MUS81*, *YEN1*, *SLX4*, *RAD1* and *MLH1* did not significantly affect the accumulation of breaks.

The simultaneous presence of broken molecules from both sides of *Alu*-PAL indicates a cleavage of the cruciform at or near its axis of symmetry. This conclusion is strongly supported by analyzing DSB formation at IRs specifically induced in the G2 phase (see below).

### Mus81/Mms4 is involved in breakage at convergently transcribed palindromes

**Figure 4.**
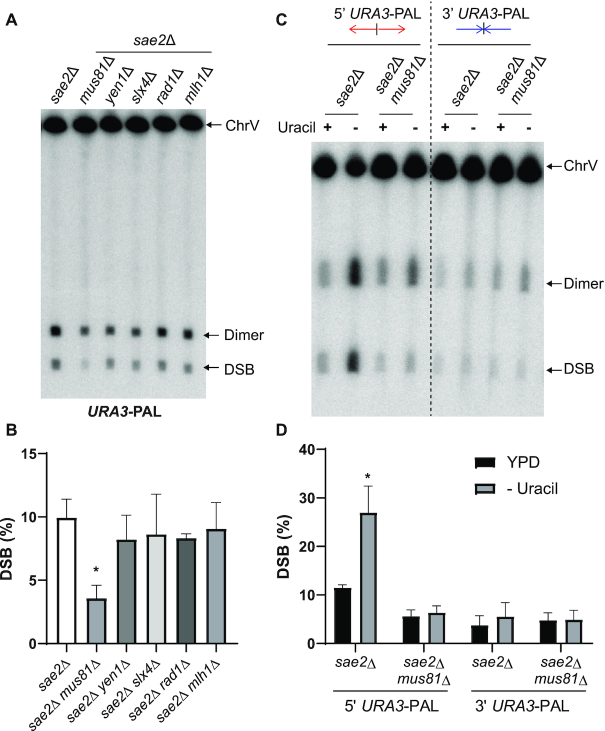
Role of Mus81/Mms4 on DSB formation at *URA3*-PAL. (**A**) Representative DSB detection at *URA3*-PAL in strains deleted for the indicated SSE. Detection has been carried out as described in Figure 1A and B. Chromosomes were separated by CHEF for 28 h. (**B**) Quantification of DSBs presented in (A). Values are the mean of at least three independent biological replicates ± standard deviation (SD); * *P*< 0.001. (**C**) Representative DSB detection at 5′ and 3′ *URA3*-PAL in *sae2*Δ and *sae2*Δ *mus81*Δ in presence (+) and absence (-) of uracil in the growth media. Detection has been carried out as described in Figure 1A and B. Chromosomes were separated by CHEF for 26 h. (**D**) Quantification of DSBs presented in (C). Values are the mean of at least three independent biological replicates ± standard deviation (SD); * *P*< 0.0001.

Among all perfect palindromes analyzed, *URA3*-PAL exhibited the strongest fragility in both WT and *sae2*Δ strains (Figure 1B). GCR rates and DSB frequencies remained unchanged upon *YEN1*, *SLX4*, *RAD1* or *MLH1* deletion in *sae2*Δ strains (Figure 4A and Supplementary Figure S3B). However, deficiency in Mus81 led to a modest, ∼2-fold, but reproducible reduction in GCRs (Supplementary Figure S3B) and DSBs induced by *URA3*-PAL (Figure 4B). Similarly, the GCR rate was ∼2-fold lower in *sae2*Δ *mus81*Δ than in *sae2*Δ at *HIS4*-PAL (Supplementary Figure S4C). This effect was specific to *URA3-* and *HIS4*-PAL since chromosomal fragility in *Alu*- and *IS50*-PAL strains was not affected in Δ*mus81* (Supplementary Figure S3A and C). The notable difference between these palindromes is that *URA3-* and *HIS4*-PAL consist of genes with active promoters located at the center of the IRs (from here on, we refer to this orientation by *5′URA3*-PAL and *5′HIS4*-PAL). This suggests that transcription can be a modifier of palindrome fragility and susceptibility for Mus81/Mms4 attack. Interestingly, the breakage potential of these two transcriptionally active palindromes is different when cells are propagated in YPD media: 30% (WT) and 10% (*sae2*Δ) at *5′URA3*-PAL vs 8% (WT) and 4% (*sae2*Δ) at *5′HIS4*-PAL (Figure 1B and C). Since breakage and repair might interfere with gene expression (54), we quantified the relative amount of mRNA expressed by the pairs of *URA3* and *HIS4* genes in *sae2*Δ *mus81*Δ. We found that the expression level of *URA3* pair is 3.5-fold higher than *HIS4* pair (Supplementary Figure S4A). This reinforces the idea that transcription at *URA3*-PAL fosters DSBs formation.

To assess the role of transcription on 5′*URA3*-PAL fragility, we analyzed the GCR rate and DSB level upon uracil starvation. It is known that in this condition *URA3* expression is induced (55). RT-qPCR analysis confirmed that the transcription level of *URA3* IRs was stimulated by a factor of 2.5 in the absence of uracil (Supplementary Figure S4A). The GCR rate in *sae2*Δ showed a ∼7.5-fold increase upon uracil starvation and deletion of *MUS81* in *sae2*Δ led to a significant ∼5-fold decrease (Supplementary Figure S4B). Consistently, DSB detection at 5′*URA3*-PAL in *sae2*Δ showed a significant increase in breakage level when the strains were grown in the absence of uracil (Figure 4C and D). In *sae2*Δ *mus81*Δ, DSB levels were similar in presence or absence of uracil (Figure 4C and D). Taken together, these results show that transcription-related DSBs are strongly dependent on Mus81/Mms4 and indicate that the complex attacks a structure whose formation is driven by transcription.

To gain insight into transcriptionally driven secondary structure formation, we analyzed the impact of transcription orientation at *URA3*-PAL. We flipped the orientation of the *URA3* genes so that transcription is directed towards the center of symmetry to create the palindrome referred to as 3′*URA3*-PAL. In this construct, *sae2*Δ strains exhibited a much lower DSB level (∼5%) compared to *5*′*URA3*-PAL (∼10%). In contrast to 5′*URA3*-PAL, neither transcription stimulation nor *MUS81* deletion had a profound impact on DSB levels at 3′*URA3*-PAL (Figure 4C and D). Uracil starvation induced a small but significant, 3-fold increase in GCRs in both *sae2*Δ and *sae2*Δ *mus81*Δ (2.9 and 2.7-fold, respectively) at *3*′*URA3*-PAL. This increase did not, however, translate into an increase in DSB level. Deletion of *MUS81* reduced the GCR rates by only 1.5- and 1.7-fold in the presence and the absence of uracil, respectively (Supplementary Figure S4B).

To confirm that substrate formation for Mus81/Mms4 cleavage at divergently transcribed palindromes is not restricted to *URA3*-PAL, we performed a similar analysis using the other transcribed palindrome, *HIS4*-PAL. *HIS4* induction was achieved by growing cells in medium lacking histidine and supplemented with 3-amino-1,2,4-triazole (3-AT). 3-AT is a competitive inhibitor of an enzyme involved in histidine production and is known to increase *HIS4* expression (56). We confirmed that growing cells in this medium stimulated the expression of the two *HIS4* genes (Supplementary Figure S4A). Similar to 5′*URA3*-PAL, in *sae2*Δ strains, *HIS4* induction led to a 4-fold increase in GCRs induced by 5′*HIS4*-PAL. Transcription-related GCRs were significantly decreased, by 2-fold, in *sae2*Δ *mus81*Δ compared to *sae2*Δ. GCRs rates were not impacted by *HIS4* induction at 3′*HIS4*-PAL in *sae2*Δ and *sae2*Δ *mus81*Δ (1.3- and 1.7-fold increase, respectively). Deletion of *MUS81* reduced the GCR rates by only 1.7- and 1.4-fold in the presence and the absence of histidine, respectively (Supplementary Figure S4C). Overall, these results are highly consistent with the fragility observed at *URA3*-PAL.

We concluded that the fragility potential at transcribed palindromes resulting from Mus81/Mms4 attack is mainly governed by both the level and the orientation of transcription.

### Cruciform extrusion and subsequent DSB formation can occur independently of replication

**Figure 5.**
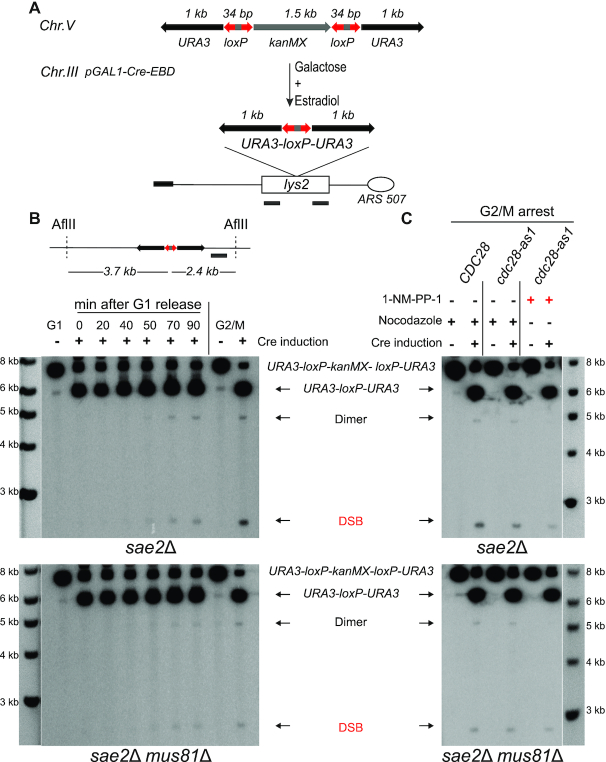
Analysis of DSB formation through the cell cycle in the conditional *URA3*-quasi palindrome. (**A**) Diagram of the conditional *URA3*-QP system. *URA3* genes (black arrows) are separated by the *kan*MX gene (gray arrow) flanked by *loxP* sites (red double arrowhead and gray triangle). This construct, *URA3-loxP-kan*MX*-loxP-URA3*, is located on chromosome V. The construct, *pGAL1-Cre-EBD*, is integrated into the *LEU2 locus* on chromosome III. *kan*MX excision leaves a 34 bp single quasi-palindromic *loxP* site with an *AT*-rich spacer. The resulting sequence, *URA3-loxP-URA3*, is a palindrome separated by 8 bp (gray bar). (**B**) DSB formation during the cell cycle in *sae2*Δ and *sae2*Δ *mus81*Δ. Cells were arrested in G1 (with α-factor) or G2/M (with nocodazole) prior to Cre-EBD expression. After 3h of incubation in media containing galactose, G1 arrested cells were released in fresh media and cells were collected at different timepoints, indicated by minutes after G1 release, for DSB analysis. G2/M arrested cells were collected after Cre expression. Genomic DNA embedded in agarose plugs was digested with *Afl*II. The position of the resulting quasi-palindrome (*URA3-loxP-URA3*) relative to *Afl*II restriction site is represented. Southern hybridization was performed using a *LYS2*-specific probe located on the ARS-proximal side of the IR (black bar). The DNA ladder, highlighted with ethidium bromide prior Southern blot hybridization, with indicated sizes is shown on the left. (**C**) Analysis of DSB formation in G2 phase in *sae2*Δ and *sae2*Δ *mus81*Δ upon Cdc28 inactivation. DSB analysis was performed like in (B). Cells were arrested in G2/M either with nocodazole in *CDC28* or *cdc28-*as1 or with 1-NM-PP-1 inhibitor in *cdc28-*as1. The DNA ladder, highlighted with ethidium bromide prior Southern blot hybridization, with indicated sizes is shown on the right.

Here we aimed to determine at which stage of the cell cycle MRX/Sae2-independent and Mus81/Mms4-mediated DSBs at IRs occur. Because DSB formation at a given phase of the cell cycle can be concealed by the existence of pre-formed DSBs, we built a *URA3*-based Cre-*lox*P conditional quasi-palindrome system. The strains contain 1 kb 5′*URA3*-IRs separated by a 1.5 kb *kanMX* gene flanked by *lox*P sites (*URA3-loxP-kan*MX*-loxP-URA3*, Figure 5A and Supplementary Figure S5A). A long spacer between repeats greatly compromises the potential to adopt a secondary structure and generate DSBs. Hence, the advantage of this system is that the pre-existing populations of DSBs and dimer intermediates are absent prior to the creation of the quasi-palindrome. The second component of the system is Cre recombinase fused in frame with the estradiol-binding domain (EBD) under the control of a *GAL1* promoter. The fusion construct is integrated into the *LEU2* locus on chromosome III (57). Upon induction with galactose and estradiol, the functional Cre recombinase is expressed, which then excises the *kanMX* leaving a single, 34 bp, palindromic *loxP* site with an 8 bp spacer (Supplementary Figure S5A). Thus, expression of the Cre recombinase and subsequent *kanMX* cassette loss lead to the creation of a *URA3*-QP8 (*URA3-loxP-URA3*). By inducing Cre expression, and thus *URA3*-QP8 formation, we followed the appearance of DSB intermediates in G1 (cells arrested with α-factor), S (cells released from G1 arrest) and G2/M (cells arrested with nocodazole) phases in *sae2*Δ and *sae2*Δ *mus81*Δ strains. The cell cycle stage was determined by monitoring cellular morphology and by flow cytometry (Supplementary Figure S5B).

DSBs were revealed using an ARS-proximal probe upon digestion of agarose embedded chromosomal DNA with *Afl*II (Figure 5B, top panel). The size of the fragment corresponding to DSBs occurring at *URA3*–8QP was expected to be 2.4 kb. In addition, a dimer of 4.8 kb was expected because of the hairpin-capped nature of the break. In uninduced conditions (-), a 7.6 kb fragment corresponding to *URA3-loxP-kanMX*-*loxP-URA3* was the predominant species highlighted and no DSBs were observed. Upon Cre induction (+), we observed the predominance of a 6.1 kb fragment devoid of *kanMX* cassette that corresponds *URA3-loxP-URA3*.

DSB analysis in *sae2*Δ showed that DSBs started to accumulate between 40 and 50 min (late S phase) after the cells were released from G1. DSB levels were even more pronounced at 70 and 90 min post-release (G2/M). In *sae2*Δ *mus81*Δ, DSBs appeared with the same dynamics, though the DSB level was lower upon *MUS81* deletion (Figure 5B bottom panel and Supplementary Figure S5C). These data show that DSBs at *URA3*-QP8 are generated by Mus81/Mms4 and another SSE after the bulk of DNA replication and suggest the DSBs accumulate in G2. Recently, Ivanova *et al.*, 2020 demonstrated that hard-to-replicate sequences such as G4 structures can be replicated when chromosome segregation has begun, in anaphase. This can explain accumulation of DSBs 90 min after α-factor release (58). However, breaks are also induced in nocodazole-arrested cells, excluding the contribution of replication. The fact that a high level of DSBs is observed in nocodazole-arrested cells strongly suggests that the breaks occur during the G2 phase. Interestingly, inverted dimers were observed in G2-arrested cells suggesting that they can result from DNA synthesis of the broken molecule outside of S-phase.

We also analyzed the symmetry of the breaks occurring at *URA3*-QP8 by cutting DNA with *Bsr*GI and probing for the telomere-proximal side of the break (Supplementary Figure S5D). This revealed accumulation of DSBs at 50, 70 and 90 min after G1 release and also in nocodazole-arrested cells. This confirms the two-ended nature of the breaks at *URA3*-QP8 and proves that cruciform resolution is the mechanism by which DSBs are generated. These data provide direct evidence that DSBs are driven by cruciform formation and resolution in G2.

### Cdk regulates Mus81-mediated breaks in G2 but not the third pathway for the cruciform resolution

To further support a replication-independent break formation at IRs, we used another way to arrest cells in G2, prior to Cre induction. We used the conditional hypomorphic *cdc28*-as1 allele that encodes a form of Cdc28^Cdk1^ that can bind the nonhydrolyzable ATP analog 1-NM-PP1. Treatment of cells harboring the *cdc28*-as1 allele with low doses of 1-NM-PP1 results in a specific inhibition of Cdc28 and causes cells to arrest in G2 (59).

DSB detection revealed that breaks are formed in *sae2*Δ in both nocodazole- and 1-NM-PP1-arrested cells (Figure 5C, top panel), confirming that breaks indeed occur in G2 phase. However, 1-NM-PP1-treated *cdc28-*as1 cells showed a decrease in DSB level, compared to the nocodazole-treated cells. Since Cdc28 potentiates Mus81/Mms4 (60,61), the decrease in break level reflects the inactivity of Mus81/Mms4 in 1-NM-PP1-treated *cdc28-*as1 cells. Indeed, DSB analysis in *sae2*Δ *mus81*Δ revealed that inactivation of Cdc28 did not further decrease DSB formation (Figure 5C, bottom panel and Supplementary Figure S5E). Strikingly, the dimer formation in 1-NM-PP1-treated *cdc28-*as1 cells was strongly decreased, if not entirely absent, in both *sae2*Δ and *sae2*Δ *mus81*Δ (Figure 5C). This suggests that one or several factors involved in hairpin-capped break duplication are under the control of Cdc28.

Overall, these data allow three main conclusions to be drawn: (i) breaks at *URA3*-QP8 are the result of a symmetrical cleavage of a cruciform structure by Mus81/Mms4 and a putative SSE, (ii) this cleavage occurs independently of DNA replication and (iii) MRX/Sae2- and Mus81/Mms4-independent breaks are perpetrated by a yet unknown nuclease whose activity is upregulated in G2, but is not under control of the Cdc28 kinase.

## DISCUSSION

**Figure 6.**
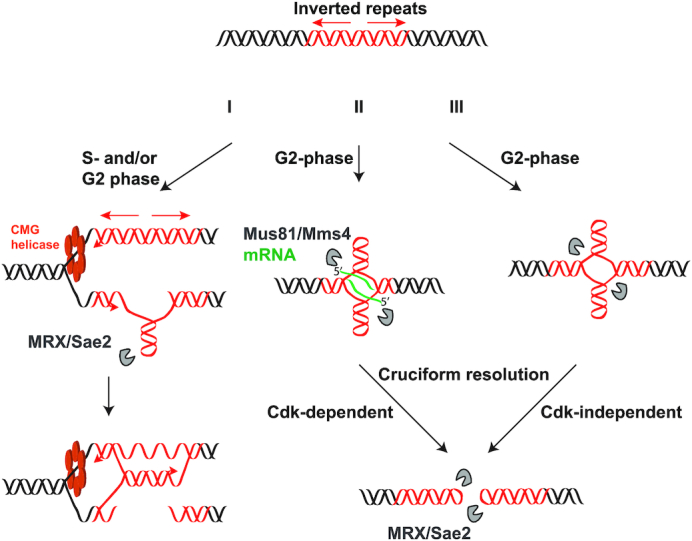
Model for DSB formation at palindromic sequences during the cell cycle. See ′Discussion’ for details.

In this study, we have investigated the role of SSEs in generating chromosome breaks at palindromic sequences in yeast. We uncovered three distinct pathways by which secondary structures can initiate DSBs and promote chromosomal instability. Very specific structural characteristics of inverted repeats define the nuclease attack mediated by MRX/Sae2 and Mus81/Mms4. Moreover, we obtained direct evidence for cruciform formation and resolution, a pathway that operates for all types of inverted repeats and which involves an unknown nuclease. A model for DSB formation at perfect and interrupted palindromes is presented in Figure 6.

### MRX/Sae2 plays a dual role in the instability of palindromes

We revealed a specific role for MRX/Sae2 in DSB generation at IRs with spacers from 0 to 8 bp. Remarkably, strong replication arrest occurs at IRs prone to MRX/Sae2 cleavage. Therefore, it is likely that stable hairpins formed on ssDNA-containing lagging strand template are attacked by the complex (Figure 6I). These breaks are repaired using the sister chromatid as a template and do not generate GCRs, thus explaining the absence of decrease in GCR rates upon *SAE2* deletion (Table 1). We also noticed that the WT strain with the most fragile palindrome (*URA3*-PAL) exhibited pink color when propagated on YPD, which indicates partial inactivation of *ADE2* expression, likely due to ongoing breakage and repair. These observations are in favor of recurrent DSB formation and restoration of palindromic sequence since palindromes are stably maintained and viability is not affected in these strains.

Even though MRX/Sae2 cleaves replication-born hairpins that stall fork progression, *SAE2* deletion does not lead to a stronger fork arrest (Figure 2F). This suggests that (i) fork pausing imposed by secondary structures at palindromes is a surmountable barrier and that (ii) MRX/Sae2 attack on hairpins occurs later, thus generating a two-ended DSB, which is consistent with the mode of action of SbcCD (25). Overall, the mechanism of MRX/Sae2-mediated breaks at palindromic sequences demonstrated in this study is very similar to the mechanism of palindrome breakage by SbcCD in bacteria described in pioneering studies of Leach and colleagues (1). MRN has also been implicated in DSB formation at palindromes in fission yeast (3,4). Thus, MRX/Sae2 DNA-structure processing properties at IRs are evolutionary conserved and conceivably, MRN/CtIP in mammals possesses the same avidity for cleaving secondary structures.

MRX/Sae2 is responsible for ∼50% of total DSBs occurring at palindromes. MRX-Sae2-independent breaks occur symmetrically and yield dimers, which is indicative of cruciform resolution and the hairpin-capped nature of the break. Thus, similar to the breaks induced at *Alu*-QP12 (28), hairpin-capped DSBs triggered at perfect palindromes require the Mre11 endonuclease activity for opening and resection.

### RPA safeguards IR stability according to the spacer length

We showed that DSB level decreases as the spacer length increases. However, DSB levels remained steady as the spacer length increased from 9 to 12 bp (Figure 2A and B). Why MRX/Sae2 does not target secondary structures formed at *Alu*-QP with >8 bp spacer is intriguing since Mre11 has the ability to cleave loops as long as 30 nt *in vitro* (62). Upon downregulation of RPA, *Alu*-QPs9–10 behaved like perfect palindromes in terms of DSB frequency, MRX/Sae2 attack, and the ability to arrest replication forks (Figure 2D–F). Hence, the apparent absence of MRX/Sae2 effect on DSB formation at *Alu*-QPs9–10 is likely due to the decreased stability and/or probability of formation of secondary structures with loops >8 nt. We propose that RPA affects both processes. When the loop size reaches 9 nt, RPA might directly destabilize hairpin structures or participate in recruiting proteins involved in DNA unwinding. For example, RPA stimulates Sgs1 unwinding activity (63) and *sgs1* mutants show an increase in both GCRs and DSB formation at *Alu*-QP12 (6) as well as an increase in recombination induced by a quasi-palindrome with 10 bp spacer (35). The scenario where RPA plays a specific role in preserving stability of quasi-palindromes is strengthened by the fact that RPA depletion augmented MRX-Sae2-dependent DSB formation by up to 20-fold at quasi-palindromes versus only 4-fold at the perfect palindrome. Binding of RPA to ssDNA loops ≤ 8 nt seems unlikely. However, a defect in RPA binding during DNA replication may lead to uncoupling between the lagging and leading DNA strand synthesis and result in a greater ssDNA exposure, thereby producing favorable conditions for hairpin formation. Indeed, instability of *Alu*-QP12 and *Alu*-QP100 in replication defective mutants prone to ssDNA accumulation, such as polymerase δ, is dramatically increased (6,28). The fact that DSB levels were similar between the perfect and quasi-palindromes in TET-*RFA2* (∼40%) argues in favor of the same mechanism increasing secondary structure formation and DSB potential, i.e. presence of unprotected ssDNA leading to hairpin formation. This is in agreement with RPA having a protective role against hairpin formation.

### Mus81/Mms4 targets preferentially transcribed palindromes

We demonstrated that breakage at perfect palindromes composed of actively transcribed *URA3* or *HIS4* genes is partially dependent on Mus81/Mms4. Transcription initiated near the center of symmetry, but not at the extremities, enhances susceptibility of palindromes to breakage, either by stimulating cruciform extrusion by creating local, negative DNA supercoiling and/or by generating a substrate targetable by Mus81/Mms4. It is well-established that *in vitro* Mus81/Mms4 prefers DNA substrates that have a free 5′ end over closed substrates such as perfect 4-way junctions (53). It is possible that the presence of an RNA molecule (specifically providing an unannealed 5′ end) at the DNA secondary structure promotes cleavage by Mus81/Mms4 (Figure 6II). The fact that breakage levels in *sae2*Δ *mus81*Δ at 5′*URA3*-PAL and in *sae2*Δ at nontranscribed palindromes (*Alu*- and *IS50*-PAL) are approximately the same, favors the idea that Mus81/Mms4 recognizes a branched DNA/RNA structure instead of transcription simply driving cruciform extrusion. Using a conditional quasi-palindrome system, we also demonstrated that Mus81/Mms4 attacks cruciform structures, likely comprising RNA molecules, in G2 stage.

Several groups reported an involvement of Mus81/Mms4 in generation of breaks at hairpin/cruciform-forming repeats (30,32,33). It is worth noting that the common feature of the repeats investigated in these studies is *AT*-richness. Poly-AT sequences are poorly bound by histones (64–66), which generates nucleosome-depleted regions where transcription is often initiated (67–69). In light of our data, we suggest that transcription taking place at *AT*-rich repeats signals Mus81/Mms4 attack.

### Cruciform extrusion and resolution are G2-mediated events

Cruciform extrusion *in vivo* in *E. coli* on plasmids has been reported in several studies (70,71) and cruciform extrusion has been proposed to occur in eukaryotic cells. For example, a replication-independent model for cruciform formation and cleavage was proposed to explain mechanism of t(11;22) translocation (72,73). However, experimental support for cruciform extrusion occurring in a chromosomal context was lacking. Using a novel conditional quasi-palindrome assay, we provide here direct evidence that *in vivo* conditions are sufficient to support cruciform extrusion in a chromosomal context in budding yeast (Figure 5). We show that susceptibility of palindromic sequences to breakage emanates in a timely manner, with cruciform extrusion and resolution occurring independently of replication, in G2 phase. It remains to be determined if cruciform resolution in G2 happens because the activity of the SSEs targeting the cruciform structure peaks in G2 and/or conditions of torsional strain leading to cruciform extrusion are met specifically in G2.

Mus81/Mms4-independent breaks appear with the same kinetic as the Mus81-induced DSBs, suggesting that the putative enzyme instigating the breaks is subject to the same regulation but is not subject to Cdc28 control. Strikingly, when we addressed the genetic dependency of DSBs at *Alu*-PAL, none of the known SSEs emerged as solely responsible for generating hairpin-capped breaks. Importantly, our conclusion is unequivocal since it is based on the physical detection of the chromosomal breaks, which is the most stringent criteria to assess the involvement of SSEs in DSB formation. We propose that a discrete pathway involving a putative nuclease is responsible for generating the MRX/Sae2 and Mus81/Mms4-independent DSBs (Figure 6III). Although we cannot completely exclude it, we do not favor the scenario where known SSEs play a redundant role in cruciform resolution. This premise is supported by the fact that inactivation of Cdc28, which is involved in the positive (Mus81, Slx4 (74,75)) and negative (Yen1 (75,76)) regulation of several nucleases, did not affect DSB formation. We also noticed that triple nuclease knockout strains grow poorly and quickly accumulate suppressors. Therefore, to address redundancy in cruciform cleavage adequately, special approaches, such as the use of auxin-regulatable conditional alleles (77), are required. Experiments are underway to identify the factor(s) involved in the proposed third pathway.

## DATA AVAILABILITY

Flow cytometry data were deposited with Flow Repository under ID FR-FCM-Z39K.

## Supplementary Material

gkab168_Supplemental_File
