# Supplementary material for: Structural parameters of palindromic repeats determine the specificity of nuclease attack of secondary structures
Source: Nucleic Acids Res. 2021 Mar 27;49(7):3932–47. doi: 10.1093/nar/gkab168 (PMC8053094; doi:10.1093/nar/gkab168)
Supplement: gkab168_Supplemental_File [file gkab168_supplemental_file.pdf]

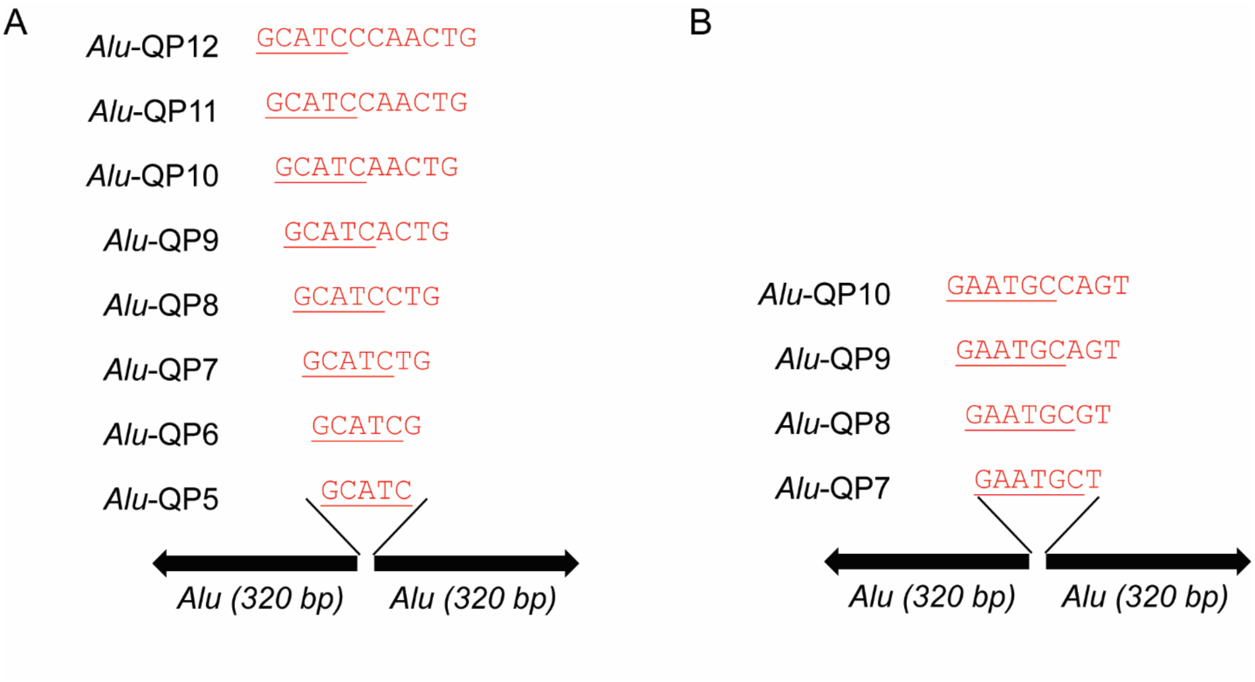

Figure S1. Asymmetrical spacers inserted at the center of *Alu* palindromes. Related to figure 1.

The sequence of the spacer inserted into *Alu*-PAL to create *Alu*-QP used in this study is presented in red. The spacer consists of the *Sfa*NI (A) or *Bsm*I (B) restriction site (underlined sequence) plus random nucleotides.

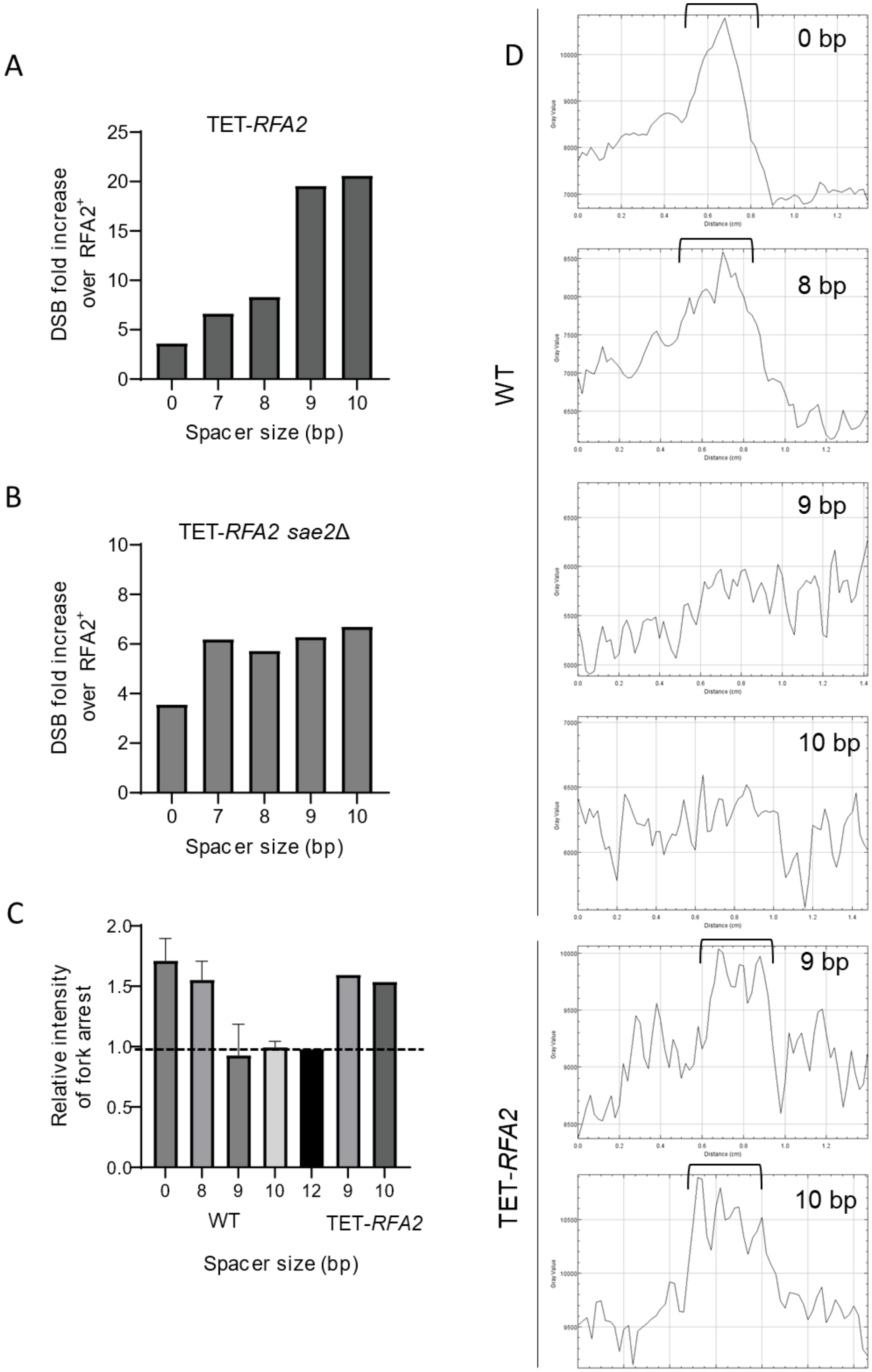
A
TET-RFA2
DSB fold increase over RFA2+
25
20
15
10
5
0
0
7
8
9
10
Spacer size (bp)
B
TET-RFA2 sae2Δ
DSB fold increase over RFA2+
10
8
6
4
2
0
0
7
8
9
10
Spacer size (bp)
C
Relative intensity of fork arrest
2.0
1.5
1.0
0.5
0.0
0
8
9
10
12
9
10
WT
TET-RFA2
Spacer size (bp)
D
WT
TET-RFA2
0 bp
8 bp
9 bp
10 bp
9 bp
10 bp

Figure S2. Effect of RPA downregulation on DSB formation at *Alu*-PAL and *Alu*-QP. Related to Figure 2.

A. and B. Data show the fold increase in DSB level in TET-*RFA2* relative to *RFA2*$^{+}$ in *WT* (A) and *sae2*Δ (B) strains. C. Quantification of the signal corresponding to site of fork arrest relative to the signal corresponding to site of unperturbed replication (downstream from the arrest). D. Densitometry analysis of the Y arc's long arm in the WT and TET-*RFA2* strains containing *Alu*-IR with indicated spacers. The presence of fork arrest is manifested as a spike in the relative signal. The bracket depicts the zone of replication arrest. Values are the mean ± standard deviation (SD).

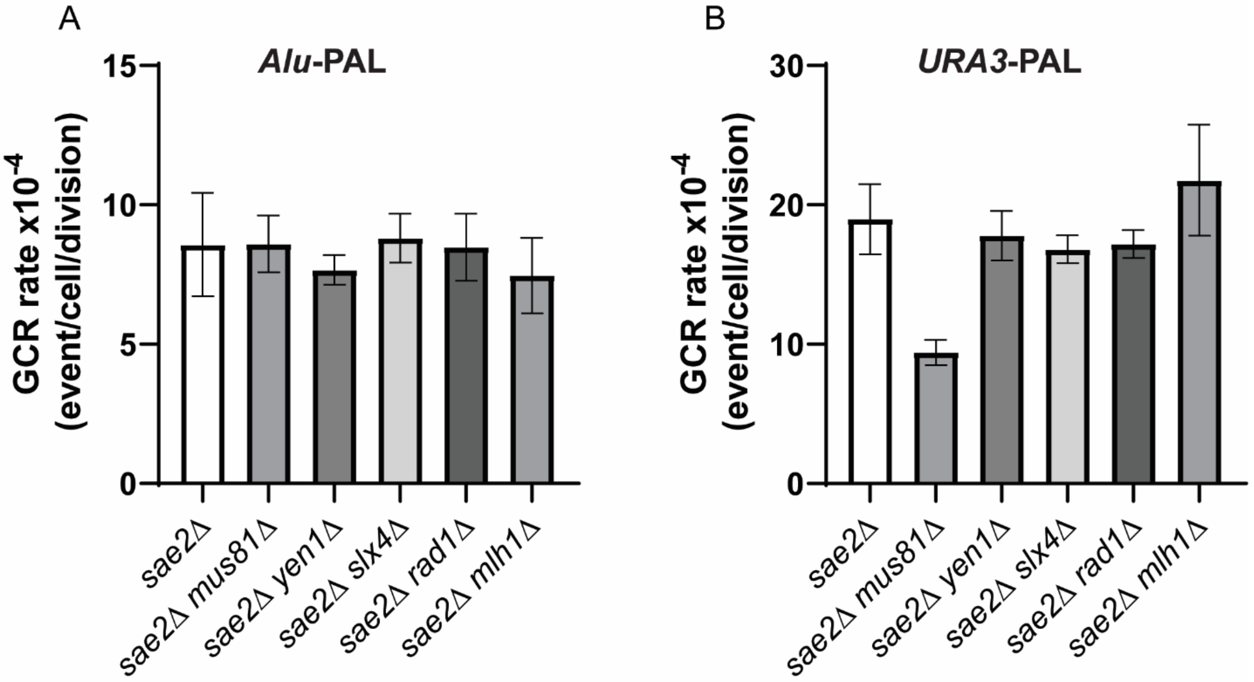

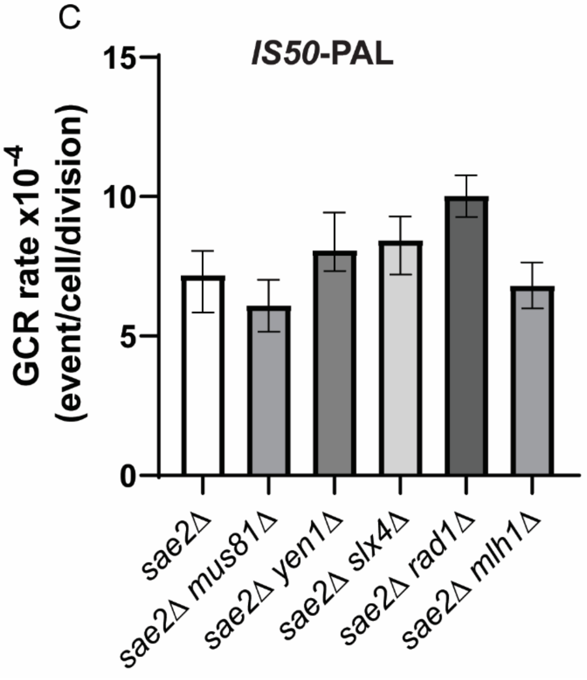

Figure S3. Effect of SSEs on palindromes-induced fragility. Related to Figure 3 and 4.

GCR rates were measured in *sae2*Δ strains deleted for either *MUS81*, *YEN1*, *SLX4*, *RAD1* or *MHL1*. A. GCR measurement at *Alu*-PAL. B. GCRs measurements at *URA3*-PAL. C. GCRs measurements at the transcribed palindrome *IS50*-PAL. Data are represented as the median value ± 95% confidence intervals.

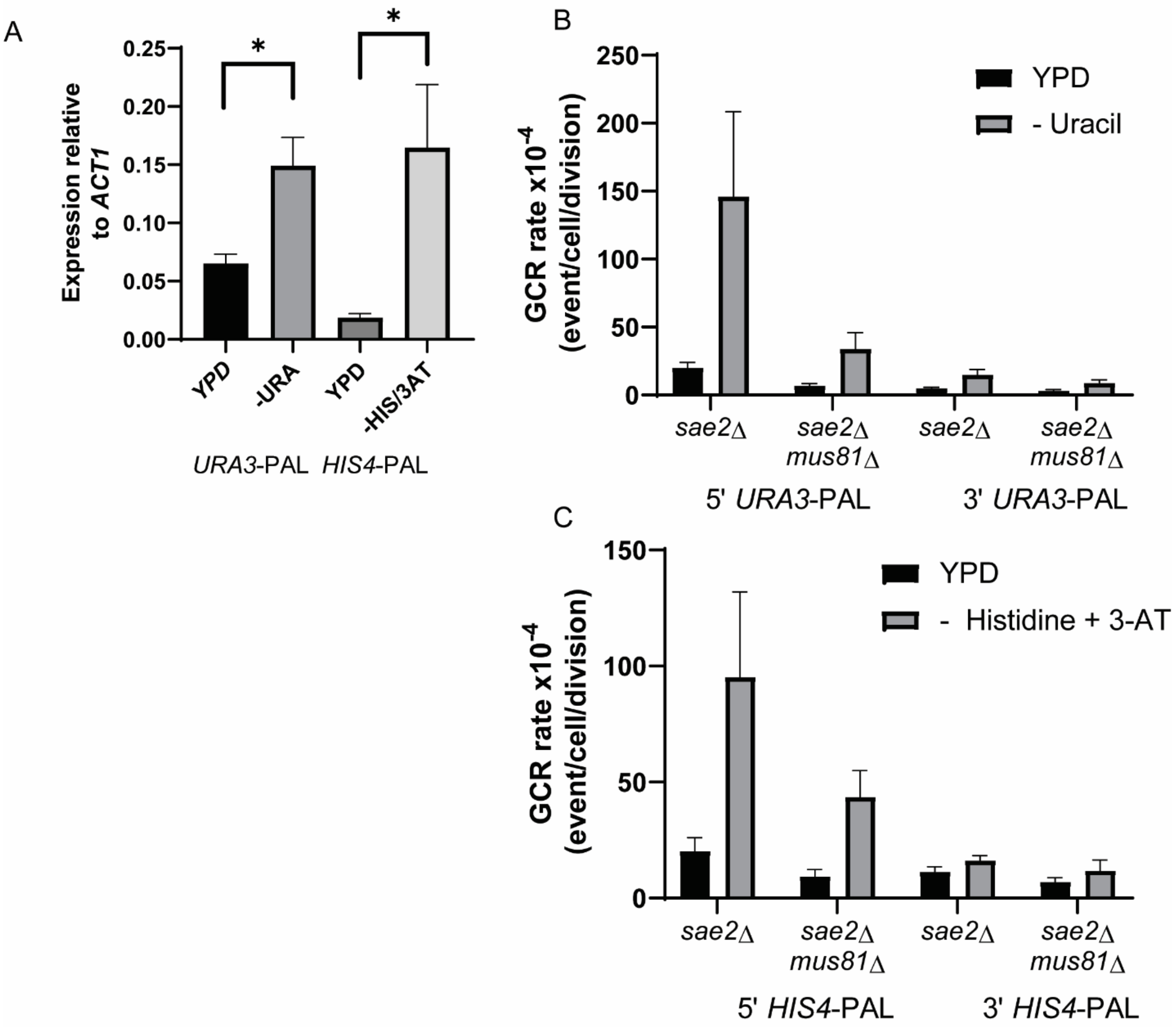

Figure S4. Effect Mus81 and transcription on GCRs induced at actively transcribed palindromes. Related to Figure 4.

A. Transcription level at *URA3* and *HIS4* measured by RT-qPCR. To analyze the effect of amino acid starvation on the transcription level, cells were cultured in YPD and uracil-depleted media to measure RNA transcript at *URA3*-PAL, and in YPD and histidine-depleted media supplemented with 3-AT to measure RNA transcript at *HIS4*-PAL. To determine the relative abundance, mRNA levels were normalized to *ACT1* and compared to the YPD control. To limit the impact of DSB formation and resection on the expression level of *URA3* and *HIS4*, the *sae2*Δ *mus81*Δ strain, in which the DSB level is minimal, was used. Data are the mean of at least 3 values ± standard deviation. Significance was estimated by two sample *t* tests. *p<0.004. B. GCR rates at 5’ and 3’ *URA3*-PAL in *sae2*Δ and *sae2*Δ *mus81*Δ in presence and absence of uracil in the growth media. C. GCR rates at 5’ and 3’ *HIS4*-PAL in *sae2*Δ and *sae2*Δ *mus81*Δ in presence of histidine or in media lacking histidine and supplemented with 3-AT. Data in B and C are represented as the median value ± 95% confidence intervals.

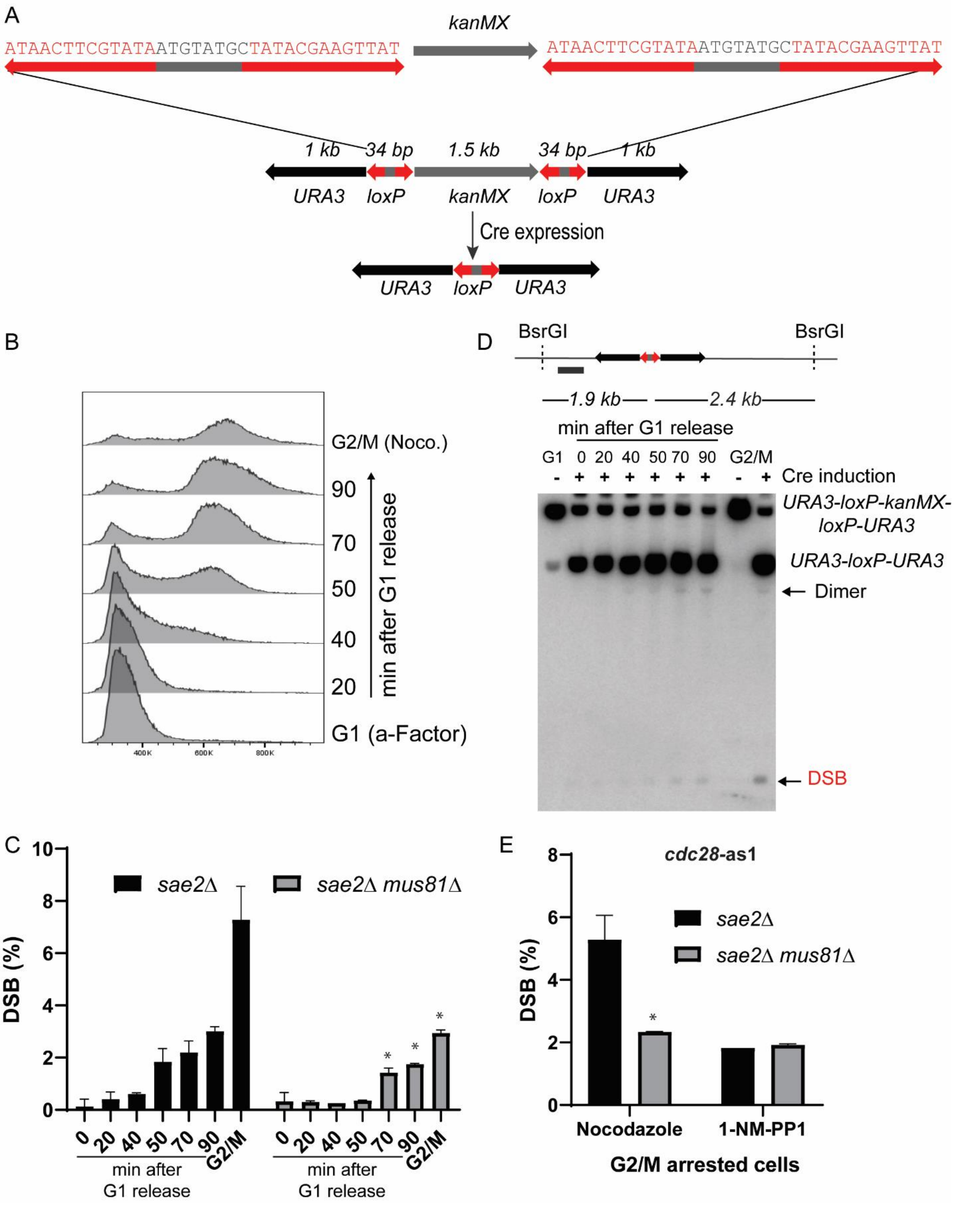
A
kanMX
ATAACTTCGTATAATGTATGCTATACGAAGTTAT
ATAACTTCGTATAATGTATGCTATACGAAGTTAT
1 kb
34 bp
1.5 kb
34 bp
1 kb
URA3
loxP
kanMX
loxP
URA3
Cre expression
URA3
loxP
URA3
B
G2/M (Noco.)
90
70
50
40
20
G1 (a-Factor)
min after G1 release
400K
600K
800K
D
BsrGI
BsrGI
1.9 kb
2.4 kb
min after G1 release
G1
0
20
40
50
70
90
G2/M
Cre induction
URA3-loxP-kanMX-loxP-URA3
URA3-loxP-URA3
Dimer
DSB
C
sae2Δ
sae2Δ mus81Δ
DSB (%)
0
2
4
6
8
10
0
20
40
50
70
90
G2/M
min after G1 release
E
cdc28-as1
sae2Δ
sae2Δ mus81Δ
DSB (%)
Nocodazole
1-NM-PP1
G2/M arrested cells

Figure S5. Conditional *URA3*-quasi palindrome to study DSB formation through the cell cycle. Related to Figure 5.

A. Diagram of the conditional *URA3*-QP system showing the *loxP* sequence. B. Representative diagram of DNA content analysis by FACS in G1 and G2/M arrested cells. Cells harboring the conditional *URA3*-QP were synchronized in G1 with alpha-factor. After Cre expression induction, cells were released into S phase. Progression through the cell cycle was analyzed at the same time points (minutes after G1 release) at which DSB analysis was performed. Cells were synchronized in G2/M with nocodazole. C. Quantification of DSBs in *sae2*Δ and *sae2*Δ *mus81*Δ presented in figure 5B. Timepoints after G1 release are indicated. G2/M corresponds to nocodazole arrested cells. Values are the mean ± standard deviation (SD). Significance was estimated by *t* test. * $p < 0.05$. D. Representative analysis of telomere-proximal DSB formation during the cell cycle in *sae2*Δ. Detection was carried out as described in Figure 5A-B. Genomic DNA embedded in agarose plugs was digested with *Bsr*GI. The position of the resulting quasi-palindrome (*URA3-loxP-URA3*) relative to the *Bsr*GI restriction site is represented. Southern hybridization was performed using a *LYS2*-specific probe located on the telomere-proximal side of the IR (black triangle) and revealed four different fragments. (i) a 5.8 kb fragment corresponding to the unbroken chromosome V containing the intact *URA3-loxP-kanMX-loxP-URA3*. This fragment is predominant when Cre is not expressed (Cre induction -). (ii) a 4.3 kb fragment corresponding to the unbroken chromosome V containing the *URA3-loxP-URA3* quasi-palindrome. This fragment becomes predominant upon Cre expression (Cre induction +). (iii) a 3.8 kb fragment corresponding to the dimer and (vi) a 1.9 kb fragment corresponding the break. E. Quantification of DSBs in *cdc28*-as1 *sae2*Δ and *cdc28*-as1 *sae2*Δ *mus81*Δ presented in figure 5C. Cells were arrested in G2/M with nocodazole or 1-NM-PP1. Values are the mean ± standard deviation (SD). Significance was estimated by *t* test. * $p < 0.05$.

Table S1. *Alu*-IR spacer length determines GCR rate

***Alu*-IR**	**GCR rate (x10^{-7})**	
	**WT**	***sae2Δ***
***Alu*-PAL**	7000 (5017-9943)[a]	7829 (6560-9459)
***Alu*-QP5**	6375 (4549-8924)	ND[b]
***Alu*-QP6**	7015 (4984-7732)	ND
***Alu*-QP7**	4449 (2853-5390)	5549 (2993-5699)
***Alu*-QP8**	3606 (4784-6584)	4603 (4062-5529)
***Alu*-QP9**	1670 (1032-4064)	2141 (1660-2722)
***Alu*-QP10**	577 (448-806)	2072 (1506-2746)
***Alu*-QP11**	603 (256-1116)	1830 (1506-2010)

[a] Numbers in parenthesis correspond to the 95% confidence interval.

[b] Not determined.

Table S2. Effect of palindromic sequences on GCRs is not sequence dependent

***Alu*-IR (BsmI spacer)**	**GCR rate (x10^{-7})**	
	**WT**	***sae2Δ***
***Alu*-QP8**	2860 (2421-3358) [a]	3951 (2992-4346)
***Alu*-QP9**	1974 (1561-2990)	3893 (3451-5490)
***Alu*-QP10**	1095 (944-1520)	3288 (2520-3632)

[a] Numbers in parenthesis correspond to the 95% confidence interval.

Table S3. Strains used in this study

Strain number	*Genotype**	*IR as referred to in the text*
**KT849**	*lys2::Alu-core*	
**YKL719**	*lys2::Alu-SwaI*	*Alu*-PAL
**YKL3210**	*lys2::Alu-SfanI-12bp*	*Alu*-QP12
**YKL3243**	*lys2::Alu-SfanI-11bp*	*Alu*-QP11
**YKL3241**	*lys2::Alu-SfanI-10bp*	*Alu*-QP10
**YKL3361**	*lys2::Alu-SfanI-9bp*	*Alu*-QP9
**YKL3209**	*lys2::Alu-SfanI-8bp*	*Alu*-QP8
**YKL3206**	*lys2::Alu-SfanI-7bp*	*Alu*-QP7
**YKL3238**	*lys2::Alu-SfanI-6bp*	*Alu*-QP6
**YKL3304**	*lys2::Alu-SfanI-5bp*	*Alu*-QP5
**YKL4074**	*lys2::Alu-BsmI-10bp*	*Alu*-QP10
**YKL4070**	*lys2::Alu-BsmI-9bp*	*Alu*-QP9
**YKL4066**	*lys2::Alu-BsmI-8bp*	*Alu*-QP8
**YKL4062**	*lys2::Alu-BsmI-7bp*	*Alu*-QP7
**YKL733**	*lys2 ::Alu-SwaI Δsae2*	*Alu*-PAL
**YKL3290**	*lys2::Alu-SfanI-12bp sae2::hphMX*	*Alu*-QP12
**YKL3288**	*lys2::Alu-SfanI-11bp sae2::hphMX*	*Alu*-QP11
**YKL3286**	*lys2::Alu-SfanI-10bp sae2::hphMX*	*Alu*-QP10
**YKL3364**	*lys2::Alu-SfanI-9bp sae2::hphMX*	*Alu*-QP9
**YKL3282**	*lys2::Alu-SfanI-8bp sae2::hphMX*	*Alu*-QP8
**YKL3987**	*lys2::Alu-SfanI-7bp sae2::hphMX*	*Alu*-QP7
**YKL4090**	*lys2::Alu-BsmI-10bp sae2::hphMX*	*Alu*-QP10
**YKL4086**	*lys2::Alu-BsmI-9bp sae2::hphMX*	*Alu*-QP9
**YKL4082**	*lys2::Alu-BsmI-8bp sae2::hphMX*	*Alu*-QP8
**YKL4078**	*lys2::Alu-BsmI-7bp sae2::hphMX*	*Alu*-QP7
**YKL3994**	*lys2 ::Alu-SwaI rfa2::TET-RFA2:kanMX4*	*Alu*-PAL
**YKL4022**	*lys2::Alu-SfanI-11bp rfa2::TET-RFA2:kanMX4*	*Alu*-QP11
**YKL4018**	*lys2::Alu-SfanI-10bp rfa2::TET-RFA2:kanMX4*	*Alu*-QP10
**YKL4014**	*lys2::Alu-SfanI-9bp rfa2::TET-RFA2:kanMX4*	*Alu*-QP9
**YKL4010**	*lys2::Alu-SfanI-8bp rfa2::TET-RFA2:kanMX4*	*Alu*-QP8
**YKL4006**	*lys2::Alu-SfanI-7bp rfa2::TET-RFA2:kanMX4*	*Alu*-QP7
**YKL4026**	*lys2 ::Alu-PAL sae2::hphMX rfa2::TET-RFA2:kanMX4*	*Alu*-PAL
**YKL4058**	*lys2::Alu-SfanI-12bp sae2::hphMX rfa2::TET-RFA2:kanMX4*	*Alu*-QP12
**YKL4054**	*lys2::Alu-SfanI-11bp sae2::hphMX rfa2::TET-RFA2:kanMX4*	*Alu*-QP11
**YKL4050**	*lys2::Alu-SfanI-10bp sae2::hphMX rfa2::TET-RFA2:kanMX4*	*Alu*-QP10
**YKL4046**	*lys2::Alu-SfanI-9bp sae2::hphMX rfa2::TET-RFA2:kanMX4*	*Alu*-QP9

**YKL4042**	*lys2::Alu-SfanI-8bp sae2::hphMX rfa2::TET-RFA2:kanMX4*	*Alu*-QP8
**YKL4038**	*lys2::Alu-SfanI-7bp sae2::hphMX rfa2::TET-RFA2:kanMX4*	*Alu*-QP7
**YKL1261**	*lys2::Alu-SwaI mre11::kanMX4*	*Alu*-PAL
**YKL1369**	*lys2::Alu-SwaI sae2::hphMX mus81::kanMX4*	*Alu*-PAL
**YKL2610**	*lys2::Alu-SwaI sae2::hphMX yen1::kanMX4*	*Alu*-PAL
**YKL1395**	*lys2::Alu-SwaI sae2::hphMX slx4::natMX*	*Alu*-PAL
**KT1255**	*lys2::Alu-SwaI sae2::TRP1 rad1::kanMX*	*Alu*-PAL
**YKL2607**	*lys2::Alu-SwaI sae2::hphMX mlh1::URA3*	*Alu*-PAL
**YKL854**	*lys2::IS50-PAL*	*IS50*-PAL
**YKL918**	*lys2::IS50-PAL sae2::TRP1*	*IS50*-PAL
**YKL2613**	*lys2::IS50-PAL sae2::TRP1 mus81::kanMX4*	*IS50*-PAL
**YKL2619**	*lys2::IS50-PAL sae2::TRP1 yen1::kanMX4*	*IS50*-PAL
**YKL2629**	*lys2::IS50-PAL sae2::TRP1 slx4::natMX*	*IS50*-PAL
**KT1259**	*lys2::IS50-PAL sae2::TRP1 rad1::kanMX*	*IS50*-PAL
**YKL2616**	*lys2::IS50-PAL sae2::TRP1 mlh1::URA3*	*IS50*-PAL
**YKL776**	*lys2::5'URA3-PAL*	*URA3*-PAL
**YKL936**	*lys2::5'URA3-PAL sae2::TRP1*	*URA3*-PAL
**YKL1229**	*lys2::5'URA3-PAL sae2::TRP1 mus81::kanMX4*	*URA3*-PAL
**YKL2663**	*lys2::5'URA3-PAL sae2::TRP1 yen1::kanMX4*	*URA3*-PAL
**YKL1360**	*lys2::5'URA3-PAL sae2::TRP1 slx4::natMX*	*URA3*-PAL
**KT1263**	*lys2::5'URA3-PAL-SwaI sae2::hphMX rad1::kanMX*	*URA3*-PAL
**YKL2650**	*lys2::5'URA3-PAL-SwaI sae2::TRP1 mlh1::URA3*	*URA3*-PAL
**KT905**	*lys2::3'URA3-PAL sae2::TRP1*	*3'URA3*-PAL
**KT917**	*lys2::3'URA3-PAL sae2::TRP1 mus81::natMX*	*3'URA3*-PAL
**YKL2869**	*lys2::5'HIS4-PAL sae2::TRP1 his4::natMX*	*5'HIS4*-PAL
**KT925**	*lys2::5'HIS4-PAL sae2::TRP1 mus81::kanMX4 his4::natMX*	*5'HIS4*-PAL
**KT1218**	*lys2::3'HIS4-PAL sae2::hphMX his4::natMX*	*3'HIS4*-PAL
**KT1224**	*lys2::3'HIS4-PAL sae2::hphMX mus81::kanMX4 his4::natMX*	*3'HIS4*-PAL
**YKL1024**	*lys2::URA3-loxP-kanMX-loxP-URA3 sae2::TRP1 leu2-3,112::GAL-Cre-EBD*	*Conditional URA3*-QP
**YKL1053**	*lys2::URA3-loxP-kanMX-loxP-URA3 sae2::TRP1 mus81::natMX leu2-3,112::GAL-Cre-EBD*	*Conditional URA3*-QP
**YKL1109**	*lys2::URA3-loxP-kanMX-loxP-URA3 sae2::TRP1 leu2-3,112::GAL-Cre-EBD cdc28::cdc28-as1*	*Conditional URA3*-QP
**KT1383**	*lys2::URA3-loxP-kanMX-loxP-URA3 sae2::TRP1 mus81::kanMX leu2-3,112::GAL-Cre-EBD cdc28::cdc28-as1*	*Conditional URA3*-QP

* All the strains share the same genetic background: *MATα bar1Δ his7-2 trp1Δ ura3Δ leu2-3,112 ade2Δ lys2Δ cup1Δ yhr054cΔ cup2Δ V34205::ADE2lys2::IR V29616::CUP1*.

Table S4. Primers used in this study

Name	Sequence	Purpose
ku-alu51	CCTCGGCCTCCCAAAGTGCTGGGATTACAGGCGTGAG CCACCGCGCCCGGCCGAGCTCGTTTTCGACACTGG	To insert CORE between Alu-IRs
ku-alu-31	CCTCCCAAAGTGCTGGGATTACAGGCGTGAGCCACC GCGCCCGGCCTCCTTACCATTAAGTTGATC	
Alu-SwaI	GCTGGGATTACAGGCGTGAGCCACCGCGCCCGGCCA TTTAAATGGCCGGGCGCGGTGGCTCACGCCTGTAATC CCAGCA	To replace core with the spacer (blue sequence for perfect palindrome and red for quasi-palindromes)
Alu-SfaNI-12bp	AAAGTGCTGGGATTACAGGCGTGAGCCACCGCGCCC GGCCGCATCCCAACTGGGCCGGGCGCGGTGGCTCAC GCCTGTAATCCCAGCACTTT	
Alu-SfaNI-11bp	AAAGTGCTGGGATTACAGGCGTGAGCCACCGCGCCC GGCCGCATCCAACTGGGCCGGGCGCGGTGGCTCACG CCTGTAATCCCAGCACTTT	
Alu-SfaNI-10bp	AAAGTGCTGGGATTACAGGCGTGAGCCACCGCGCCC GGCCGCATCAACTGGGCCGGGCGCGGTGGCTCACGC CTGTAATCCCAGCACTTT	
Alu-SfaNI-9bp	AAAGTGCTGGGATTACAGGCGTGAGCCACCGCGCCC GGCCGCATCACTGGGCCGGGCGCGGTGGCTCACGCC TGTAATCCCAGCACTTT	
Alu-SfaNI-8bp	AAAGTGCTGGGATTACAGGCGTGAGCCACCGCGCCC GGCCGCATCCTGGGCCGGGCGCGGTGGCTCACGCCT GTAATCCCAGCACTTT	
Alu-SfaNI-7bp	AAAGTGCTGGGATTACAGGCGTGAGCCACCGCGCCC GGCCGCATCTGGGCCGGGCGCGGTGGCTCACGCCTGT AATCCCAGCACTTT	
Alu-SfaNI-6bp	AAAGTGCTGGGATTACAGGCGTGAGCCACCGCGCCC GGCCGCATCGGGCCGGGCGCGGTGGCTCACGCCTGT AATCCCAGCACTTT	
Alu-SfaNI-5bp	AAAGTGCTGGGATTACAGGCGTGAGCCACCGCGCCC GGCCGCATCGGCCGGGCGCGGTGGCTCACGCCTGTA ATCCCAGCACTTT	
Alu-BsmI-10bp	AAAGTGCTGGGATTACAGGCGTGAGCCACCGCGCCC GGCCGAATGCCAGTGGCCGGGCGCGGTGGCTCACGC CTGTAATCCCAGCACTTT	
Alu-BsmI-9bp	AAAGTGCTGGGATTACAGGCGTGAGCCACCGCGCCC GGCCGAATGCAGTGGCCGGGCGCGGTGGCTCACGCC TGTAATCCCAGCACTTT	
Alu-BsmI-8bp	AAAGTGCTGGGATTACAGGCGTGAGCCACCGCGCCC GGCCGAATGCGTGGCCGGGCGCGGTGGCTCACGCCT GTAATCCCAGCACTTT	
Alu-BsmI-7bp	AAAGTGCTGGGATTACAGGCGTGAGCCACCGCGCCC GGCCGAATGCTGGCCGGGCGCGGTGGCTCACGCCTG TAATCCCAGCACTTT	

S2870	CTGACGACTCTCAGTTTACCAATGT	Telomere proximal probe for Alu-PAL
O3138	GAGAGGGGTACGAACTTGGTAGCGTCTCA	
S3630	CTTCTACTCTTGACACTGAATACTAC	ARS-proximal probe
O3970	TCTACTGGAACCATATTCACGGA	
S2360	TTATCTACCAAACGGTGACTG	Telomere proximal probe for URA3-PAL
O2600	GTTTCAACCTCCTTTGGAAC	
URA3-rt5	GAACGTGCTGCTACTCATC	RT-qPCR at URA3-PAL
URA3-rt3	ACTCCAGTAATTCCTTGGTG	
HIS4-rt5	GTTGTTGTGGAAGAGAACGG	RT-qPCR at HIS4-PAL
HIS4-rt3R	TTGGTCGACAACTAGGGTGG	
ACT1-rt5	AACCGCTGCTCAATCTTC	RT-qPCR at ACT1
ACT1-rt3R	CAATACCGGCAGATTCCAA	
